# Aptamer–Gemcitabine Conjugates with Enzymatically Cleavable Linker for Targeted Delivery and Intracellular Drug Release in Cancer Cells

**DOI:** 10.3390/ph15050558

**Published:** 2022-04-30

**Authors:** Jianjun Qi, Zihua Zeng, Zhenghu Chen, Cole Nipper, Xiaohui Liu, Quanyuan Wan, Jian Chen, Ching-Hsuan Tung, Youli Zu

**Affiliations:** 1 Department of Pathology and Genomic Medicine, Houston Methodist Hospital, 6565 Fannin Street, Houston, TX 77030, USA; jqi@houstonmethodist.org (J.Q.); zzeng@houstonmethodist.org (Z.Z.); zchen2@houstonmethodist.org (Z.C.); cnipper@houstonmethodist.org (C.N.); 101013182@seu.edu.cn (X.L.); wanq@ccf.org (Q.W.); jchen@houstonmethodist.org (J.C.); 2 Department of Radiology, Molecular Imaging Innovations Institute, Weill Cornell Medicine, New York, NY 10021, USA; cht2018@med.cornell.edu

**Keywords:** aptamer–drug conjugates, enzymatically cleavable linker, controlled intracellular drug release, high gemcitabine payload, targeted drug delivery, cell-specific chemotherapy

## Abstract

Gemcitabine is a chemotherapeutic used clinically to treat a variety of cancers. However, because it lacks tumor cell specificity, gemcitabine may cause off-target cytotoxicity and adversely impact patients. To impart cancer cell specificity to gemcitabine and improve its therapeutic efficacy, we synthesized a unique aptamer–drug conjugate that carries a high gemcitabine payload (three molecules) via a dendrimer structure and enzymatically cleavable linkers for controlled intracellular drug release. First, linker–gemcitabinedendrimer–linker–gemcitabine products were produced, which had significantly lower cytotoxicity than an equimolar amount of free drug. Biochemical analysis revealed that lysosomal cathepsin B protease rapidly cleaved the dendritic linkers and released the conjugated gemcitabine as a free drug. Subsequently, the dendrimer–linker–gemcitabine was coupled with a cell-specific aptamer to form aptamer–gemcitabine conjugates. Functional assays confirmed that, under aptamer guidance, aptamer–gemcitabine conjugates were selectively bound to and then internalized by triple-negative breast cancer cells. Cellular therapy studies indicated that the aptamer–gemcitabine conjugates potentiated cytotoxic activity to targeted cancer cells but did not affect off-target control cells. Our study demonstrates a novel approach to aptamer-mediated targeted drug delivery that combines a high drug payload and an enzymatically controlled drug release switch to achieve higher therapeutic efficacy and fewer off-target effects relative to free-drug chemotherapy.

## 1. Introduction

Traditional systemic chemotherapeutics non-specifically kill any proliferating cells, including those that are non-cancerous. Gemcitabine is a common and well-studied chemotherapeutic used as a monotherapy or in combination with other agents to treat many cancers, including pancreatic, bladder, breast, non-small cell lung, and ovarian cancers [1]. Like many non-targeted chemotherapeutic agents, gemcitabine has dose-limiting side effects related to its off-target activity, including gastrointestinal toxicity, myelosuppression, rash, dyspnea, and fever [1]. Ligand-targeted therapeutics can minimize these side effects by facilitating the targeted delivery of cytotoxic agents to cancer cells, though highly specific targeting ligands with a robust safety profile are required.

Oligonucleotide aptamers are promising candidates for targeted chemotherapy delivery systems. Generated through a recursive selection process termed the systematic evolution of ligands by exponential enrichment (SELEX), aptamers are short segments of DNA or RNA that form unique 3D structures and bind targets with an antibody-like specificity and affinity [2]. They are chemically synthesized, readily modifiable using simple chemistry, and inherently non-immunogenic [3]. The flexibility of aptamer platforms and the relative ease of aptamer selection make them a promising tool for cancer diagnosis and treatment. Due to the fact that oligonucleotide aptamers are smaller than analogous antibodies, they penetrate solid tumors relatively rapidly and effectively [4]. Numerous aptamer-based therapeutics have been developed, including growth-factor inhibitors [5], aptamer–drug conjugates [6], aptamer-labeled nanoparticles [7,8,9], and aptamer-labeled liposomes [10,11].

Despite many developments in aptamer therapeutic platforms, only a few are approved for clinical use [12]. One barrier hindering the translation of aptamer therapeutics from the lab to the clinic is unpredictable in vivo behavior. This unpredictability often stems from the specific techniques used for aptamer drug loading. DNA-intercalating therapeutics suffer from instability and poor tunability due to GC-region scarcity. Nanoparticles functionalized via aptamer labeling may have nephrotoxicity or poor tissue penetrance. Directly conjugated aptamer–drug pairs are often limited by a 1:1 aptamer–drug stoichiometry. Thus, more reliable aptamer–drug loading techniques are needed.

**Figure 1:**
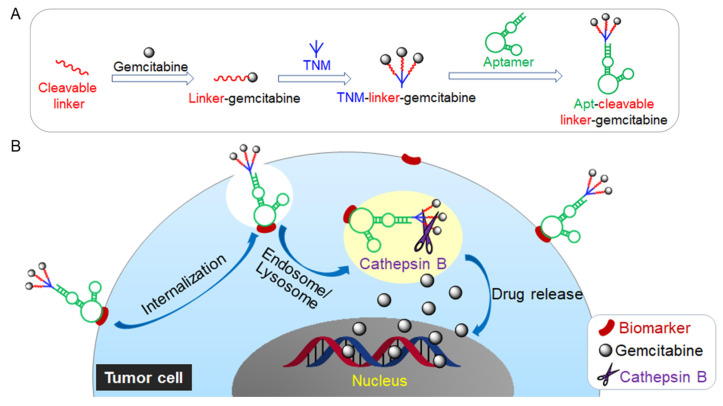
(**A**) Schematic illustration of the construction of Aptamer, enzymatically cleavable peptide Linker, and tri-Gemcitabine (Apt–cL–triGemcitabine) drug conjugate. (**B**) Targeted chemotherapy by Apt–cL–triGemcitabine with enzymatically controlled lysosomal drug release.

To address these shortcomings, we present a unique aptamer–drug conjugate that incorporates a trifurcated Newkome-type monomer (TNM) to improve drug loading capacity with enzymatically cleavable peptide linkers for endosomal drug release and an ssDNA aptamer to target triple-negative-breast-cancer (TNBC) cells. Aptamer-mediated cell binding allows the intracellular delivery and subsequent release of multiple molecules of gemcitabine (Figure 1). The liberation of gemcitabine, in turn, provokes tumor-cell death without off-target side effects.

## 2. Results

### 2.1. Synthesis and Validation of TNM Dendrimer-Cleavable Linker-Gemcitabine Drug Structures

**Figure 2:**
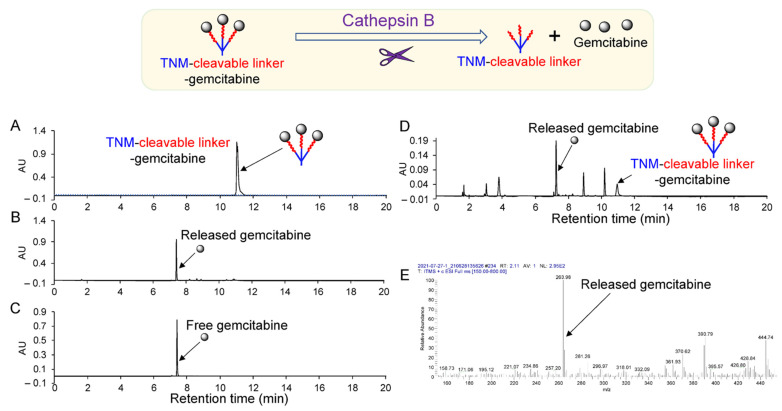
Characterization of enzymatically controlled drug release. (**A**) HPLC chromatogram of synthetic linker–gemcitabineTNM–linker–gemcitabine products. (**B**) Cathepsin B-mediated free drug release from linker–gemcitabineTNM–linker–gemcitabine. (**C**) Control chromatogram of gemcitabine drug. (**D**) Cell lysate-mediated free drug release from TNM–linker–gemcitabine. (**E**) Mass spectrum identification of released free drugs from linker–gemcitabineTNM–linker–gemcitabine after cathepsin B treatment.

TNM dendrimer-cleavable linker–gemcitabine drug (TNM–linker–gemcitabine) products were synthesized in two phases (Materials and Methods; Scheme S1, Figures S1–S14. To characterize the enzymatically controlled release of gemcitabine, TNM–linker–gemcitabine was treated with lysosomal cathepsin B protease at 37 °C for 1 h, and the resultant products were analyzed by high-performance liquid chromatography (HPLC). Cathepsin B treatment-induced gemcitabine’s release from linker–gemcitabineTNM–linker–gemcitabine was seen as a new peak in the HPLC chromatogram at a retention time identical to free drug control (Figure 2A–C). For further characterization, cultured TNBC cells (MDA-MB-231) were harvested, and fresh cell lysate was prepared as a source of cellular lysosomal enzymes. linker–gemcitabineTNM–linker–gemcitabine was incubated with cell lysates at room temperature (RT) for 1 h, and the resultant products were analyzed by HPLC chromatogram. A distinct peak of free gemcitabine was detected along with a small remaining peak of linker–gemcitabineTNM–linker–gemcitabine (Figure 2D). To verify that the released gemcitabine was unaltered by the process of conjugation and enzymatic release, the molecular weight of the released drug was determined. Mass spectroscopy confirmed that the released gemcitabine molecules were intact and unaltered (Figure 2E).

To determine biostability, linker–gemcitabineTNM–linker–gemcitabine was incubated at 37 °C for 1 h under different pH conditions and the resultant drug release was quantified. HPLC analysis revealed that linker–gemcitabineTNM–linker–gemcitabine is stable at a physiological pH of 7.0 and lysosomal pH (5.0); only 4.1% and 4.6% of the drug was released after 1 h, respectively (Figure S15). In addition, linker–gemcitabineTNM–linker–gemcitabine was incubated with inactivated cathepsin B at 37 °C for 1 h at pH 5.0. In the absence of active cathepsin B, minimal gemcitabine release (6%) was detected by HPLC (Figure S16). Notably, careful optimization of the reaction conditions was necessary to perform this analysis as dimethylformamide, a solvent required for the drug release assay, inhibits cathepsin B at high concentrations (Figure S17). These findings confirm the controlled enzymatic release of free gemcitabine from linker–gemcitabineTNM–linker–gemcitabine.

### 2.2. Formulation of Apt–cL–triGemcitabine for Targeted Chemotherapy

**Figure 3:**
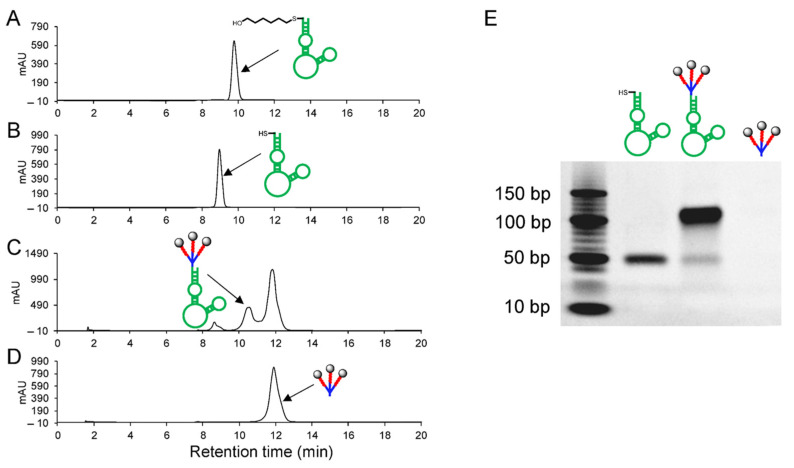
Preparation and characterization of Apt–cL–triGemcitabine. (**A**) HPLC chromatogram of synthetic aptamer sequence. (**B**) Deprotected aptamer sequence. (**C**) Formed Apt–cL–triGemcitabine conjugates. (**D**) linker–gemcitabineTNM–linker–gemcitabine products. (**E**) Gel electrophoresis of deprotected aptamer sequence, Apt–cL–triGemcitabine conjugates, and linker–gemcitabineTNM–linker–gemcitabine products (not visible on gel).

For targeted TNBC cell delivery, linker–gemcitabineTNM–linker–gemcitabine was conjugated to the ssDNA aptamer PDGC21-T to create Apt–cL–triGemcitabine (Scheme S2). Deprotection of 5-ThioMC6-D–aptamer (Figure 3A), performed in TCEP (tris(2-carboxyethyl)phosphine) aqueous solution, was confirmed by a decrease in retention time in HPLC analysis (Figure 3B). The deprotected aptamer sequence was mixed with linker–gemcitabineTNM–linker–gemcitabine at 4 °C for 4 days. The resultant conjugate products were analyzed by HPLC, and a new peak consistent with Apt–cL–triGemcitabine products was detected between the peaks of the aptamer and the linker–gemcitabineTNM–linker–gemcitabine, respectively (Figure 3B–D). Apt–cL–triGemcitabine products were purified by semi-preparative reversed-phase HPLC and examined by gel electrophoresis. Apt–cL–triGemcitabine products have an increased molecular weight compared to the unconjugated aptamer control sequence (Figure 3E). Notably, linker–gemcitabineTNM–linker–gemcitabine was not visible on the gel but was detected using HPLC. Due to the fact that this coupling reaction occurred between two molecules of high molecular weight, the reaction proceeded slowly and required four to six days to yield sufficient products.

### 2.3. PDGC21-T Aptamer Probe Specifically Targets TNBC Cells

**Figure 4:**
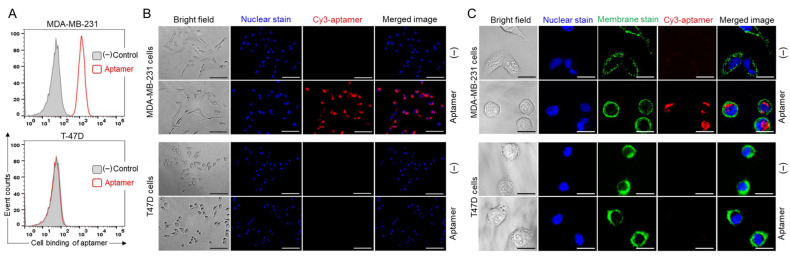
Specific cell binding of PDGC21-T aptamers. (**A**) Flow cytometry analysis of cell binding by Cy3-labeled aptamer probes to MDA-MB-231 (TNBC) and T47D (non-TNBC) cells. (**B**) Fluorescent microscopy of cell binding by Cy3-labeled aptamer probes. For visualization, cell nuclei were stained with Hoechst dye. Scale bar = 100 µm. (**C**) Confocal microscopy revealed aptamer internalization into MDA-MB-231 (TNBC) cells but not T47D (non-TNBC) cells. For visualization of individual cells, nuclei and membranes were stained with Hoechst dye and DiO Cell-Labeling solution, respectively. Scale bar = 25 µm.

To validate the specificity of the PDGC21-T aptamer cell binding, cultured TNBC (MDA-MB-231) and non-TNBC (T47D) cells were harvested and treated with Cy3-labeled PDGC21-T aptamers for 30 min. Flow cytometry analysis revealed that aptamer probes selectively bound MDA-MB-231 cells with high affinity (Kd = 10.04 ± 1.89 nM) and did not react with T47D cells (Figure 4A). Probes consisting of random ssDNA sequences were used as a background control in the cell-binding assay. For further validation, treated cells were examined using fluorescent microscopy. PDGC21-T aptamers selectively stained MDA-MB-231 cells but not T47D cells (Figure 4B). These findings support the TNBC-specific binding of the PDGC21-T aptamer and underscore its potential for guided therapy in TNBC.

To assess aptamer-mediated drug delivery, cultured cells were treated with Cy3-labeled PDGC21-T aptamers for 30 min at 37 °C and then washed to remove free aptamers. Cell nuclei and membranes were stained with Hoechst dye and DiO Cell-Labeling Solution (a lipophilic carbocyanine dye) for tracking purposes, respectively. Confocal microscopy revealed an aptamer-derived fluorescent signal within the cytoplasm of MDA-MB-231 cells that was not present within off-target T47D cells (Figure 4C), indicating specific internalization of the aptamer by targeted tumor cells.

### 2.4. TNM–Linker–Gemcitabine Products Have Minimal Cytotoxicity

**Figure 5:**
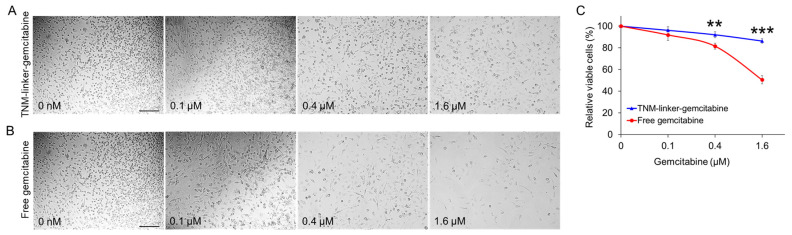
Linker–gemcitabineTNM–linker–gemcitabine has minimal cytotoxicity compared to free gemcitabine. Cultured tumor cells (MDA-MB-231) were treated for 30 min with linker–gemcitabineTNM–linker–gemcitabine (**A**) or free gemcitabine (**B**) at different concentrations as indicated. Treatment concentrations were adjusted to reflect an equimolar amount of gemcitabine. After treatment, cells were washed and cultured in fresh medium for two days. At the endpoint, cell cultures were examined and imaged under a light microscope (**A**,**B**), and cell proliferation rates were evaluated by CCK-8 proliferation assay (**C**). Scale bar = 200 µm. *p* ≤ 0.05 was considered significant. **: *p* ≤ 0.01; ***: *p* ≤ 0.001 (Student’s *t*-test).

To compare the cytotoxicity potentials of linker–gemcitabineTNM–linker–gemcitabine and free gemcitabine, cultured MDA-MB-231 cells were seeded in a 96-well plate and treated with linker–gemcitabineTNM–linker–gemcitabine or free gemcitabine at different concentrations at 37 °C for 30 min. The treatment concentrations were adjusted to reflect equimolar amounts of gemcitabine (i.e., 1:3 ratio of linker–gemcitabineTNM–linker–gemcitabine to free gemcitabine). Post treatment, drug-containing supernatant was discarded, and cells were washed twice. The treated cells were then cultured in a fresh medium for two days. At the endpoint, cell cultures were examined using light microscopy (Figure 5A), and cell proliferation rates were evaluated by CCK-8 proliferation assay (Figure 5B). Relative to free-drug treatment, linker–gemcitabineTNM–linker–gemcitabine had minimal effect on the cellular proliferation of MDA-MB-231 cells, 15% inhibition vs. 50% by free gemcitabine treatment at 1.6 µM. These findings indicate that incorporation of gemcitabine into linker–gemcitabineTNM–linker–gemcitabine reduces its cytotoxicity compared to free drug and may help eliminate non-specific chemotherapy effects during drug administration and delivery.

### 2.5. Apt–cL–triGemcitabine Conjugate Treatment Induces Apoptosis and Death of Target TNBC Cells

**Figure 6:**
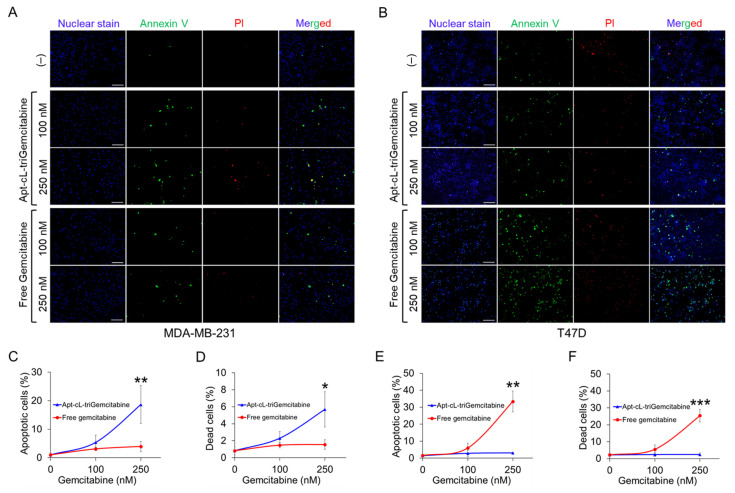
Apt–cL–triGemcitabine conjugate treatment induces apoptosis and death of targeted TNBC cells. Cultured tumor cells were treated as indicated for two days and stained with Annexin V (green fluorescence) to mark apoptotic cells, propidium iodide (PI) (red) to mark dead cells, and Hoechst dye (blue) for cell tracking purposes. (**A**) Fluorescent microscopic images of MDA-MB-231 cells (TNBC) treated with Apt–cL–triGemcitabine conjugate or free gemcitabine drug at equimolar amounts of gemcitabine, or no treatment as background baseline controls. (**B**) Fluorescent microscopic images of T47D (non-TNBC) cells with the same set of treatments. (**C**,**D**) Quantitative analysis of apoptosis and death rates in MDA-MB-231 cells post treatments as indicated. (**E**,**F**) Quantitative analysis of apoptosis and cell death rates of off-target T47D cells under the same treatment conditions. Scale bar = 200 µm. Significant differences between groups are indicated with asterisks. *p* ≤ 0.05 was considered significant. *: *p* ≤ 0.05; **: *p* ≤ 0.01; ***: *p* ≤ 0.001 (Student’s *t*-test).

To evaluate the therapeutic potential, cultured MDA-MB-231 (TNBC) and T47D (non-TNBC) cells were treated with Apt–cL–triGemcitabine conjugates or equimolar amounts of the free gemcitabine drug at 37 °C for 30 min to allow aptamer-mediated cell binding. To mimic in vivo circulation conditions, cells were washed to remove therapeutic agents and subsequently cultured for two days. At the endpoint, cells were stained with propidium iodide (PI) and Annexin V to highlight dead and apoptotic cells, respectively. For tracking purposes, cell nuclei were stained with Hoechst 33342 dye, and resultant changes in cell apoptosis and death were examined under a fluorescent microscope (Figure 6A,B). Quantitative analysis revealed that the Apt–cL–triGemcitabine conjugate treatment had greater therapeutic potential to induce apoptosis and death of targeted MDA-MB-231 cells than equimolar amounts of the free gemcitabine treatment (Figure 6C,D, Table S1). In contrast, Apt–cL–triGemcitabine conjugates showed little toxicity toward off-target T47D cells, although an equimolar free gemcitabine treatment induced significant cell apoptosis and death under the same treatment conditions (Figure 6E,F, Table S1). These findings demonstrate that, under aptamer guidance, Apt–cL–triGemcitabine conjugates specifically target cells of interest for efficient chemotherapy while simultaneously mitigating gemcitabine toxicity in off-target cells.

### 2.6. Apt–cL–triGemcitabine Conjugate Inhibits Proliferation of Targeted TNBC Cells

**Figure 7:**
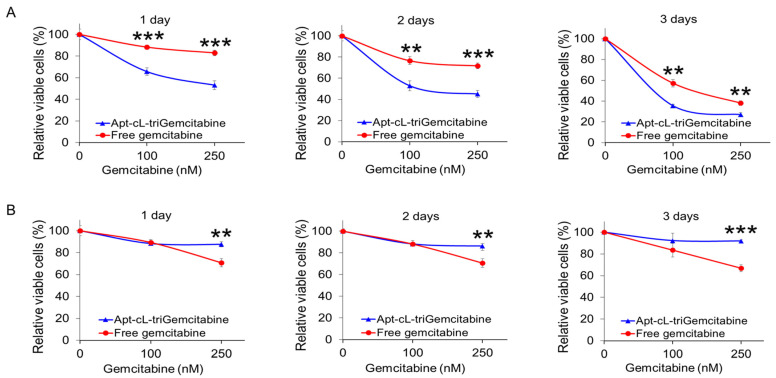
Apt–cL–triGemcitabine conjugate treatment selectively inhibits TNBC cell proliferation with few side effects on off-target cells. Cultured MDA-MB-231 (TNBC) cells (**A**) and T47D (non-TNBC) cells (**B**) were treated with Apt–cL–triGemcitabine conjugate or free drug at equimolar amounts of gemcitabine as indicated. Resultant changes in cell proliferation rates (%) were kinetically measured using a CCK-8 cell proliferation assay kit at day 1, 2, and 3 post treatment. Untreated cells were used as a background baseline control. Significant differences between groups are indicated with asterisks. *p* ≤ 0.05 was considered significant. **: *p* ≤ 0.01; ***: *p* ≤ 0.001 (Student’s *t*-test).

To evaluate cell growth effects, cultured tumor cells were treated with an Apt–cL–triGemcitabine conjugate or equimolar amounts of the free gemcitabine drug for 30 min as described earlier. Cells were then washed and subsequently cultured in a fresh medium for three days. Changes in cell proliferation were kinetically evaluated daily by a CCK-8 assay. Compared to an equimolar amount of the free gemcitabine drug, the Apt–cL–triGemcitabine conjugate treatment significantly inhibited MDA-MB-231 cell proliferation in a dose-dependent manner (Figure 7A). In contrast, Apt–cL–triGemcitabine induced significantly less cell growth inhibition compared to free gemcitabine at higher concentrations in off-target T47D cells. Notably, both MDA-MB-231 and T47D cells had a dose-dependent susceptibility to free gemcitabine drug treatment. These findings demonstrate that the Apt–cL–triGemcitabine conjugate treatment selectively inhibited proliferation in tumor cells of interest with few side effects on off-target cells.

## 3. Discussion

Here, we report a novel aptamer–drug conjugate that potentiates the therapeutic efficacy of gemcitabine in TNBC cells and reduces off-target drug cytotoxicity in non-TNBC cells. Gemcitabine, a deoxycytidine analog that disrupts dividing cells [13], is a well-studied chemotherapeutic used in the treatment of a variety of cancers. Its mechanism of action allows it to preferentially kill proliferating cell populations, including cancer. Like many non-specific chemotherapeutic agents, however, off-target cytotoxicity can result in deleterious and dose-limiting side effects. Although gemcitabine is regularly used to treat cancers, chemoresistance is common. Chemoresistance can be attributed to the altered expression of nucleoside transporter-1 (hENT1), a key receptor involved in the internalization of free gemcitabine by cancer cells [14]. Various drug delivery techniques have been employed to overcome gemcitabine resistance, such as incorporating gemcitabine into liposomes or nanoparticles [15], conjugating gemcitabine to peptide carriers [16], and incorporating gemcitabine into a G-quadruplex aptamer. Park et al. showed that the gemcitabine-modified aptamer, APTA-12, significantly improved the anti-tumor activity of gemcitabine in nucleolin-overexpressing pancreatic cancer cells [17]. In this study, we demonstrate that the Apt–cL–triGemcitabine conjugate has substantially greater anti-neoplastic activity than free gemcitabine in TNBC cells. We postulate that this improvement stems from efficient aptamer-mediated cell targeting and intracellular delivery of gemcitabine, as evidenced by aptamer internalization by TNBC cells (Figure 4C). In addition, the conjugation of gemcitabine with an enzymatically cleavable linker facilitated the controlled intracellular drug release by lysosomal cathepsin B protease (Figure 2 and Figure 3). Moreover, by incorporating TNM dendrimer structures, three gemcitabine molecules were added to each Apt–cL–triGemcitabine, promoting efficient drug delivery with a high drug payload.

The clinical utility of chemotherapeutics is constrained by the tolerability of their side effects. Due to the fact that side effects typically arise from off-target cytotoxicity, therapeutic strategies that minimize off-target activity can facilitate higher dosing and, by extension, greater therapeutic effect. Our central objective was to develop a novel chemotherapeutic with specific anti-tumor activity in TNBC cells but limited activity in healthy tissue. To this end, we hypothesized that the chemotherapeutic could be stabilized with chemical moieties that prevent extracellular drug release. In the past, the clinical adoption of aptamer therapeutics has been hindered by instability and unpredictable behavior in vivo. To prevent aptamer release of gemcitabine in circulation, we incorporated cleavable peptide linkers into the TNM dendrimer vehicle. These linkers, commonly used in antibody–drug conjugate development, are enzymatically cleaved by activated cathepsin B in lysosomes. Notably, our study reveals that, by incorporating gemcitabine into the TNM vehicle using these cleavable linkers, the cytotoxic activity of gemcitabine was markedly reduced relative to free drug (Figure 5), which may increase its drug delivery safety profile.

## 4. Materials and Methods

### 4.1. Chromatography and Mass Spectroscopy

All reagents were purchased from Sigma-Aldrich (St. Louis, MO, USA) or Tokyo Chemical Industry (Tokyo, Japan) and used without further purification. Column chromatography was performed on silica gel 60 (70–230 mesh) (Fair Lawn, NJ, USA). Analytical thin-layer chromatography was conducted on glass plates coated with silica gel 60 (F-254) (EMD-Millipore Corporation, Billerica, MA, USA). Mass spectra (ESI-MS data) were collected on an LCQ-Fleet™ Ion Trap Mass Spectrometer (Thermo, Waltham, MA, USA) and Waters Maldi SYNAPT HDMS Q-TOF Mass Spectrometer (Milford, MA, USA).

### 4.2. Synthesis and Characterization of Apt–cL–triGemcitabine Conjugate

*Synthesis of TNM-linker–gemcitabine.* The TNM–linker–gemcitabine was synthesized in two phases according to Scheme S1. In the first phase, Boc-protected linker–gemcitabine (compound **6**) was prepared using established peptide chemistry. Boc-Val-Cit-OH (compound **3**) was coupled with p-aminobenzyl alcohol (PABOH) using N-Ethoxycarbonyl-2-ethoxy-1,2-dihydroquinoline (EEDQ) to form compound **4**, followed by the reaction with 4-nitrophenyl-chloroformate to form an activated carbonate moiety (compound **5**). Compound 5 was conjugated to gemcitabine to create the Boc-protected cleavable linker peptide–gemcitabine (compound **6**). The mass spectrum of compound **6** is shown in Figure S4. 

In the second phase, Boc-protected peptide-Gemcitabine was incorporated into a TNM. TNM (compound **10**) was coupled with 6-maleimidocaproic acid in the presence of 1-[Bis(dimethylamino)methylene]-1H-1,2,3-triazolo [4,5-b]pyridinium 3-oxide hexafluorophosphate (HATU) to form compound **11**. Compound **11** was deprotected to remove tert-butoxy, forming compound **12**. Compound **12** was activated with HATU completely to form active ester compound **13**. Dropwise addition of compound **13** to deprotected peptide–gemcitabine in Dimethylformamide (DMF) and N-Methylmorpholine (NMM) formed TNM–peptide–gemcitabine (compound **14**). The mass spectrum of compound **14** is shown in Figures S11 and S12.

*Synthesis and characterization of Apt–cL–triGemcitabine.* Detailed methods are described in the supplementary materials.

### 4.3. Drug Release from TNM-Linker-Gemcitabine

*Cathepsin B-mediated Release of free Gemcitabine drug*: Dithiothreitol (DTT) solution (30 mM) was prepared by adding 4.5 mg of DTT to 1 mL of sodium acetate buffer (pH = 5.0, ~0.1 M). Then, 1 µL (0.45 µg) of cathepsin B solution (molecular weight: 35,000, purchased from EMD Millipore, Burlington, MA, USA) was added to 40 µL of DTT solution and the mixture was incubated at 37 °C for 15 min. Subsequently, 2 µL (6.5 µg) of linker–gemcitabineTNM–linker–gemcitabine stock solution (in DMF) was added to the activated cathepsin B solution and incubated for 1 h at 37 °C. The reaction solution was analyzed by HPLC with a reversed-phase column.

*Cell Lysate Preparation*: Two-day cultured MDA-MB-231 cells were trypsinized, washed twice with PBS (phosphate buffered saline), and quantified. Next, 1 × 10^7^ cells were resuspended in an Eppendorf tube with 200 µL PBS. Cells were sonicated in a 4 °C water bath in a 30 s off/on cycle for 10 min. After sonication, cells were centrifugated at 10,000× *g* for 60 min. The supernatant was transferred to a clean tube and stored at −20 °C for further use.

*Cell Lysate-mediated Release of Gemcitabine*: A 10 µL aliquot of DTT solution was added to 30 µL of cell lysate and incubated for 15 min at 37 °C. After activation of the cell lysate solution, 1 µL (0.32 µg) of linker–gemcitabineTNM–linker–gemcitabine stock solution (diluted ×10) was added to the cell lysate and incubated for 1 h at 37 °C. The reaction solution was analyzed by HPLC with a reversed-phase column.

### 4.4. HPLC Analysis

*HPLC Operating Conditions for Aptamer–linker–gemcitabineTNM–linker–gemcitabineCoupling*: HPLC separations were performed using a PRP-1 C18 column (4.1 × 150 mm, 5 µm, Hamilton, Reno, NV, USA) and the Varian 920-LC liquid chromatograph (Walnut Creek, CA, USA). The reaction was monitored using gradient HPLC, and analytes were detected using an ultraviolet absorbance detector at a wavelength of 260 nm. The mobile phase consisted of solvent A (100 mM of triethylammonium acetate (TEAA) aqueous solution) and solvent B (5% of mobile A in acetonitrile) with a flow rate of 1.0 mL/min. The mobile phase composition was maintained at 100% solvent A for 3 min before changing linearly to 0% solvent A (3–18 min), which was held for 3 min (18–21 min). Subsequently, the mobile phase was returned to its initial conditions over 3 min (21–24 min) and maintained at 100% solvent A for 2 min (24–26 min) to equilibrate the column.

*HPLC Operating Conditions for Purification*: Semi-preparative HPLC was performed using a PRP-1 C18 column (10 × 250 mm, 10 µm, Hamilton, Reno, NV, USA) and the Hitachi L-710 liquid chromatograph with an L-7455 diode array detector (Chiyoda-ku, Tokyo, 100-8280, Japan). Purification was conducted using gradient HPLC and analytes were detected using an ultraviolet absorbance detector at a wavelength of 260 nm. The mobile phase consisted of solvent A (100 mM of TEAA aqueous solution) and solvent B (5% of mobile A in acetonitrile) at a flow rate of 3.0 mL/min. The mobile phase composition was maintained at 100% solvent A for 4 min before changing linearly to 0% solvent A (4–24 min), which was held for 4 min (24–28 min). Subsequently, the mobile phase was returned to its initial conditions over 3 min (28–30 min) and maintained at 100% solvent A for 2 min (30–32 min) to equilibrate the column.

*HPLC Operating Conditions for Drug Release*: HPLC separation was performed using a Phenomenex Luna C18 column (150 × 4.60 mm, 5 µm, Phenomenex, Torrance, CA, USA) and the HPLC quaternary solvent manager pump (Waters Corporation, Milford, MA, USA) with a 2998 PDA ultraviolet absorbance detector (Waters Corporation, Milford, MA, USA). Gemcitabine release was monitored using gradient HPLC and analytes were detected using an ultraviolet absorbance detector at a wavelength of 260 nm (gemcitabine) and 215 nm (decomposed compounds). The mobile phase consisted of solvent A (0.1% trifluoroacetic acid aqueous solution) and solvent B (0.05% trifluoroacetic acid in acetonitrile) at a flow rate of 1.0 mL/min. The mobile phase composition was maintained at 100% solvent A for 3 min before changing linearly to 0% solvent A (3–18 min), which was held for 3 min (18–21 min). Subsequently, the mobile phase was returned to its initial conditions over 3 min (21–24 min) and maintained at 100% solvent A for 2 min (24–26 min) to equilibrate the column. The injection volume was 20 µL.

### 4.5. Gel Electrophoresis

Thiol-PDGC21-T aptamer (0.4 µg), Apt–cL–triGemcitabine (0.5 µg), and linker–gemcitabineTNM–linker–gemcitabine (0.5 µg) were loaded onto a 10% denaturing PAGE (Polyacrylamide) gel. Separation was performed at 60 V/cm for 1.5 h in 0.5 × TBE running buffer. The gel was stained with 1× SYBR Green for 25 min and imaged using a Gel Doc™ EZ imaging system (Bio-Rad, Hercules, CA, USA).

### 4.6. Cell Lines and Cell Culture

Thr MDA-MB-231 (human TNBC) and T47D (human non-TNBC) cell lines were purchased from the American Type Culture Collection (Manassas, VA, USA) and tested negative for mycoplasma. The MDA-MB-231 cells were cultured in Dulbecco’s Modified Eagle’s Medium (DMEM) (Corning, Manassas, VA, USA), and T47D cells were cultured in Roswell Park Memorial Institute (RPMI) 1640 medium (HyClone, GE Healthcare Life Sciences, South Logan, UT, USA). Both media were supplemented with 10% FBS (Atlanta Biologicals, Inc., Flowery Branch, GA, USA), 100 U/mL penicillin, and 100 µg/mL streptomycin (Gibco, Waltham, MA, USA). Cells were maintained at 37 °C in 5% CO_2_ and ≥95% humidity.

### 4.7. PDGC21-T Aptamer Probe Synthesis

The PDGC21-T aptamer sequence was previously reported as ACACCAAAATCGTCCGTTTCGTTTTAGTCCGTCTCTTTAGGGTGT. Cy3-conjugated PDGC21-T was synthesized by Integrated DNA Technologies (Coralville, IA, USA). A 100 µM stock solution of the aptamer probe was prepared with nuclease-free water and stored at −20 °C.

### 4.8. Cell-Binding Assay

*Flow Cytometry Analysis*: Two-day cultured cells (MDA-MB-231 and T47D) were trypsinized and washed twice with PBS. The Cy3-PDGC21-T conjugate was heated at 95 °C for 5 min and then cooled on ice for 10 min. Cells were mixed with a Cy3-PDGC21-T probe in a binding buffer to a final concentration of 200 nM and incubated in the dark at RT for 30 min. After incubation, the cells were washed twice with a washing buffer and subjected to flow cytometry analysis with a BD LSR II cytometer (BD Biosciences, San Jose, CA, USA). Flow cytometry data were analyzed using FlowJo v10.0.7 software (FlowJo, Ashland, OR, USA).

*Fluorescent Microscopy*: MDA-MB-231 and T47D cells were cultured in black 96-well plates for 1 day and then incubated with a 200 nM of Cy3-PDGC21-T probe in s culture medium at 37 °C for 1 h. After incubation, aptamer-treated and control cells were counterstained with Hoechst 33342 dye for 5 min. The staining solution was discarded and 100 µL fresh culture medium was added to cells. Cells were examined using an Olympus IX81 fluorescence microscope (Olympus Corporation, Shinjuku City, Tokyo, Japan).

*Confocal Microscopy:* MDA-MB-231 cells and T47D cells were cultured in black 96-well plates for 1 day and then incubated with 200 nM of a Cy3-PDGC21-T probe in a culture medium at 37 °C for 30 min. The cells were washed once with PBS, and then cell membranes were counterstained with Vybrant™ DiO Cell-Labeling Solution (5 µL/mL) at 37 °C for 15 min, and nuclei were stained with Hoechst 33342 dye for 5 min. To evaluate aptamer internalization, the cells were examined using a Nikon A1 Confocal Imaging System (Nikon, Tokyo, Japan).

### 4.9. Cell Proliferation Assay

#### 4.9.1. Toxicity Evaluation of TNM-gemcitabine

*Cell treatment:* 4 × 10^3^ MDA-MB-231cells in 100 µL of DMEM culture medium were seeded into 96-well plates. Cells were incubated with synthetic TNM–gemcitabine products or free drug at equimolar amounts of gemcitabine, at final concentrations of 100 nM, 400 nM, and 1600 nM for 30 min at 37 °C. After incubation, the drug-containing supernatant was discarded, the cells were washed twice with PBS, and fresh DMEM was added. The cells were further cultured at 37 °C in 5% CO_2_ and ≥95% humidity for two days.

*CCK-8 Proliferation Assay:* Cell proliferation was quantified using the Cell Counting Kit-8 (CCK-8) assay (APExBIO Technology, Houston, TX, USA) according to the manufacturer’s instructions. Cell proliferation was evaluated one, two, or three days after drug administration. Formazan products were measured using a BioTek Synergy H4 microplate reader (Winooski, VT, USA) at a wavelength of 450 nm. All experiments were performed in triplicate, and the average absorbance standard deviation was calculated. Student’s *t*-tests were performed to assess different groups.

#### 4.9.2. Effects of Apt–cL–triGemcitabine Conjugate on TNBC Cells

*Cell Treatment:* 4 × 10^3^ cells in 100 µL of DMEM culture medium (MDA-MB-231 cells) or RPMI 1640 culture medium (T47D cells) were seeded into 96-well plates. Cells were incubated with the Apt–cL–triGemcitabine conjugate or the free gemcitabine drug at concentrations of 100 nM or 250 nM for 30 min at 37 °C. An untreated control group was prepared using the same number of cells. After incubation, the drug-containing supernatant was discarded, cells were washed twice with PBS, and fresh DMEM or RPMI 1640 culture medium was added.

*Fluorescence Microscopy*: Two days after treatment with the Apt–cL–triGemcitabine conjugate or free gemcitabine drug, MDA-MB-231 and T47D cells were stained with a mixture of PI (Invitrogen, Eugene, OR, USA), Annexin V (BD Biosciences, Franklin Lakes, NJ, USA), and Hoechst 33342 (Thermo Fischer Scientific, Waltham, MA, USA) dyes at RT for 10 min. The supernatant was discarded, cells were washed with PBS, and 100 µL of new culture medium was added. Cells were examined using an Olympus IX81 fluorescence microscope. Images of each experimental group were captured at low magnification (×100). Annexin V positive apoptosis cells and PI positive dead cells were quantified for four fields of each experiment. Mean values and standard deviations were calculated.

*CCK-8 Proliferation Assay*: Cell proliferation was evaluated one, two, or three days after drug administration using the methods prescribed previously. All experiments were performed in triplicate, and the average absorbance standard deviation was calculated. Student’s *t*-tests were performed to assess the different groups and treatment timepoints.

### 4.10. Data Analysis

Statistical analysis was performed using Microsoft Excel software. An unpaired Student’s *t*-test was used in the data analysis, and *p* < 0.05 was considered a statistically significant difference.

### 4.11. Study Design and Rationale

The goal of ligand-directed cancer therapy is to minimize the side effects related to the activity of the cytotoxic agents in normal cells while simultaneously delivering these agents at therapeutic doses to the target cells. To this end, we designed aptamer–drug conjugates to facilitate the targeted delivery of gemcitabine, an effective and well-validated chemotherapeutic, to cancer cells. To introduce a controlled drug-release switch, gemcitabine was conjugated to the carrier vehicle through an enzymatically cleavable linker (Figure 1A). This peptide linker is highly stable in blood but readily cleaved by activated cathepsin B (present in the low pH microenvironment of cell lysosomes). In addition, the linker conjugation masks the 4-NH2 group on gemcitabine, a region that contributes to its cytotoxicity [18]. To increase the drug payload, we incorporated three linker–gemcitabine molecules into a TNM. To increase the specificity for cancer cells, we conjugated the TNM–linker–gemcitabine products to PDGC21 aptamer sequences that selectively bind TNBC cells [19]. The formulated Aptamer, enzymatically cleavable Linker, and tri-Gemcitabine (Apt–cL–triGemcitabine) drug conjugate allows one aptamer to carry three gemcitabine molecules, ensures stable drug delivery, and results in an enzymatically controlled lysosomal drug release.

Under aptamer guidance, the Apt–cL–triGemcitabine conjugate will bind the TNBC cells and be rapidly internalized [20]. Within targeted cells, enzymatic cleavage of Apt–cL–triGemcitabine by lysosomal cathepsin B protease will liberate gemcitabine molecules. These freed molecules will disrupt DNA synthesis, resulting in apoptosis and the death of targeted tumor cells (Figure 1B). This drug-delivery system should have high therapeutic efficacy as it promotes the selective accumulation of gemcitabine in targeted cancer cells and hinders the non-specific uptake of gemcitabine by healthy cells.

## 5. Conclusions

In summary, this study reports a new approach for developing aptamer therapeutics that carry a high drug payload and contain an enzymatically controlled intracellular drug release switch for targeted delivery. Our in vitro studies demonstrate that the Apt–cL–triGemcitabine conjugate has higher therapeutic efficacy in cancer cells of interest and causes less off-target toxicity than a free gemcitabine drug treatment.

## Data Availability

Data are available in the article and the Supplementary Materials.

## Supplementary Materials

The following supporting information can be downloaded at: https://www.mdpi.com/article/10.3390/ph15050558/s1, Scheme S1: Synthesis of TNM-linker-Gemcitabine structure; Scheme S2: Coupling reaction of aptamer with TNM-linker-Gemcitabine structure; Figure S1: ESI mass spectrum of compound **3** in Scheme S1; Figure S2: ESI mass spectrum of compound **4** in Scheme S1; Figure S3: ESI mass spectrum of compound **5** in Scheme S1; Figure S4: ESI mass spectrum of compound **6** in Scheme S1; Figure S5: ESI mass spectrum of compound **7** in Scheme S1; Figure S6: ESI mass spectrum of compound **9** in Scheme S1; Figure S7: ESI mass spectrum of compound **10** in Scheme S1; Figure S8: ESI mass spectrum of compound **11** in Scheme S1; Figure S9: ESI mass spectrum of compound **12** in Scheme S1; Figure S10: ESI mass spectrum of compound **13** in Scheme S1; Figure S11: Maldi mass spectrum of compound **14** in Scheme S1; Figure S12: ESI mass spectrum of compound **14** in Scheme S1; Figure S13: HPLC chromatogram. purification of Apt-cL-triGemcitabine conjugate products by semi-preparative reversed-phase HPLC; Figure S14: ESI mass spectrum of byproduct. Figure S15: HPLC chromatogram of TNM-linker-Gemcitabine in different pH buffer; Figure S16: The chromatogram of TNM-linker-Gemcitabine structure post-treatment with inactive cathepsin B enzyme in pH = 5 buffer at 37 °C for 1 h; Figure S17: The chromatogram of TNM-liker-Gemcitabine structure treated with activated cathepsin B enzyme in pH = 5 buffer for 1 h at 37 °C. Table S1: Cell apoptosis and death rates two-days post treatments as indicated.
