# Supplementary material for: Aptamer–Gemcitabine Conjugates with Enzymatically Cleavable Linker for Targeted Delivery and Intracellular Drug Release in Cancer Cells
Source: Pharmaceuticals (Basel). 2022 Apr 30;15(5):558. doi: 10.3390/ph15050558 (PMC9147807; doi:10.3390/ph15050558)
Supplement: Supplementary file 1 [file pharmaceuticals-15-00558-s001.zip › pharmaceuticals-1690737-supplementary.pdf]

Supporting Information

In vitro Aptamer-gemcitabine Conjugates with Enzymatically Cleavable Linker for Targeted Delivery and Intracellular Drug Release in Cancer Cells

Jianjun Qi †[1], Zihua Zeng †[1], Zhenghu Chen[1], Cole Nipper[1], Xiaohui Liu[1], Quanyuan Wan[1], Jian Chen[1], Ching-Hsuan Tung[2], and Youli Zu *[1]

[1]Department of Pathology and Genomic Medicine, Houston Methodist Hospital, 6565 Fannin Street, Houston, Texas 77030, United States

[2]Molecular Imaging Innovations Institute, Department of Radiology, Weill Cornell Medicine, New York, NY 10021

† Co-first author, these authors contributed equally to this work

* Correspondence: yzu@houstonmethodist.org

Materials and Methods

General Information About Chemistry Experiments

All reagents were purchased from Sigma-Aldrich (St. Louis, MO, USA) or TCI (Tokyo, Japan) and used without further purification. Column chromatography was performed on silica gel 60 (70-230 Mesh; Fair Lawn, NJ, USA). Analytical thin-layer chromatography (TLC) was conducted on glass plates coated with silica gel 60 (F-254; EMD Millipore Corporation, Billerica, MA, USA); Mass spectra (ESI-MS data) were collected on an LCQ Fleet™ Ion Trap Mass Spectrometer (Thermo, Waltham, MA, USA) and The Waters Q-Tof Maldi SYNAPT Mass Spectrometer (Milford, MA, USA).

Experimental Section

Synthesis and Characterization

Tert-butyl (1-((1-amino-1-oxo-5-ureidopentan-2-yl) Amino)-3-methyl-1-oxobutan-2-yl) Carbamate (compound 3) (Yanming Wang, 2017). N-(tert-butoxycarbonyl)-L-valine (compound 1) (1.1 g, 5 mmol) was dissolved in 30 mL of dichloromethane. N-hydroxysuccinimide (624 mg, 5.4 mmol) and 1.1g (5.3 mmol) N, N'-dicyclohexylcarbodiimide were added to the reaction solution. The reaction solution was stirred at room temperature (RT) for 2 h then filtered. The filtrate was concentrated, and the residue was dissolved in 30 mL of acetonitrile and filtered. To this filtrate-containing active ester, a solution of L-citrulline (compound 2, 1.75 g, 10 mmol) was added in 0.2 N sodium bicarbonate. The reaction solution was stirred at RT overnight. The reaction solution was then filtered, and the filtrate was concentrated. The residue was acidified with 1 N hydrochloric acid to pH 1-2, extracted with ethyl acetate, dried over sodium sulfate, and concentrated to give 680 mg crude product (yield 35.9%) that did not need to be purified and was used directly in the next reaction step. MS: 374 $(M+H)^+$, 749 $(2M+H)^+$ (Figure S-1, the sample for mass spectrometer analysis was purified by TLC.) TLC: Rf=0.43 (ethyl acetate/methanol=1/1).

Preparation of Tert-butyl (1-((1-((4-(hydroxymethyl) phenyl)amino)-1-oxo-5-ureidopentan-2-yl) amino)-3-methyl-1-oxobutan-2-yl) Carbamate (compound 4) (Yanming Wang, 2017). Compound 3 (crude product) (680 mg, 1.82 mmol) was dissolved in a mixture solution of dichloromethane (18 mL) and methanol (9 mL). To this solution, we added 972 mg (3.94 mmol) of 2-ethoxy-1-ethoxycarbonyl-1,2-dihydroquinoline (EEDQ), then 4-aminobenzyl alcohol (484 mg, 3.93 mmol). The reaction solution was stirred at RT overnight. The solvent was removed by rotavapor. To the residue was added dichloromethane; the solution was shocked and then filtered to give 0.61 g of a white solid (yield 70%). This

solid did not need to be purified and was directly used in the next reaction step. TLC: Rf=0.57 (ethyl acetate/methanol=5/2). MS: 502 $(M+Na)^+$, 981 $(2M+Na)^+$ (Figure S-2, the sample for mass spectrometer analysis was purified by TLC.).

Preparation of Tert-butyl (1-((1-((4-(hydroxymethyl) phenyl) amino)-1-oxo-5-ureidopentan-2-yl) amino)-3-methyl-1-oxobutan-2-yl) Carbamate (compound 5). Compound 4 (crude product) (120 mg, 0.25 mmol) was dissolved in 2 mL of dimethylformamide. To this reaction solution, 0.2mL of N,N-diisopropylethylamine was added, then 4-nitrophenyl chloroformate (110 mg, 0.54 mmol). The reaction solution was stirred overnight at RT. It was checked by TLC when the reaction was almost over, then evaporated by rotavapor. The residue was purified by silica gel column, and eluted with ethyl acetate and acetonitrile to give 83 mg of pure product as white solid (yield 51.6%). TLC: Rf=0.5 (eluted with ethyl acetate one time, then eluted with acetonitrile twice). MS: 667 $(M+Na)^+$(Figure S-3).

Preparation of 4-(2-(2-((tert-butoxycarbonyl) amino)-3-methylbutanamido)-5-ureidopentanamido) benzyl (1-((2R,4R,5R)-3,3-difluoro-4-hydroxy-5-(hydroxymethyl)tetrahydrofuran-2-yl)-2-oxo-1,2-dihydropyrimidin-4-yl) carbamate (compound 6). Compound 5 (83 mg, 0.129 mmol) was dissolved in 4 mL of dimethylformamide. To this reaction solution, 0.4 mL of N,N-diisopropylethylamine was added, then gemcitabine (169 mg, 0.644 mmol). The reaction solution was stirred for 48 h at RT, checked by HPLC when the reaction was almost over, and then evaporated by rotavapor. The residue was dissolved in methanol, purified by silica gel column, and eluted by ethyl acetate and ethyl acetate/methanol =5/1 and 5/2 to give 79 mg of pure compound 6 (yield 79.8%). MS: 769 $(M+H)^+$, 791 $(M+Na)^+$ (Figure S-4).

Preparation of 4-(2-(2-amino-3-methylbutanamido)-5-ureidopentanamido) Benzyl (1-((2R,4R,5R)-3,3-difluoro-4-hydroxy-5-(hydroxymethyl) Tetrahydrofuran-2-yl)-2-oxo-1,2-dihydropyrimidin-4-yl) Carbamate (compound 7). Compound 6 (79 mg, 0.1 mmol) was dissolved in 0.5 mL of methanol. A 1.5 mL aliquot of trifluoroacetic acid was added to this reaction solution. The reaction solution was stirred for 1 h at RT, then evaporated by rotavapor to give compound 7. MS: 335 $(M+2H)^{2+}$, 669 $(M+H)^+$, 1337 $(2M+H)^+$ (Figure S-5).

Preparation of Di-tert-butyl 3,3'-((2-((3-(tert-butoxy)-3-oxopropoxy)methyl)-2-(6-(2,5-dioxo-2,5-dihydro-1H-pyrrol-1-yl) hexanamido) propane-1,3-diyl) bis(oxy)) Dipropionate (compound 11). 6-Maleimidocaproic acid (8, 21.1 mg, 0.1 mmol) was dissolved in dimethylformamide (1 mL) and N-methylmorpholine (0.1 mL), then added to HATU (38 mg, 0.1 mmol) and stirred for 2 h at RT, monitored by mass spectrometer, MS: 352(M+Na)+ (Figure S-6). The reaction was almost over. Then, 110 mg (0.2mmol) of compound 10 (Figure S-7) (Zihua Zeng, 2021) was added to the above solution and stirred at RT overnight. The reaction solution was evaporated by rotavapor. The residue was purified by silica gel column, and eluted with hexane and hexane/ ethyl acetate =3/1, 2/1 to give 634 mg of compound 11 (yield 90.8%). TLC: Rf=0.5 (Hexane/ ethyl acetate=1/1). MS: 721 $(M+Na)^+$ (Figure S-8).

Preparation of 3,3'-((2-((2-carboxyethoxy) methyl)-2-(6-(2,5-dioxo-2,5-dihydro-1H-pyrrol-1-yl) hexanamido) Propane-1,3-diyl) bis(oxy)) Dipropionic Acid (compound 12). Compound 11 (70 mg, 0.1 mmol) was dissolved in a mixture solvent of trifluoroacetic acid (475 μL) and water (25 μL) at RT and stirred for 1 h. It was checked by HPLC and mass spectrometer to confirm the reaction was complete. Then the reaction solution was evaporated by rotavapor. The residue did not need to be purified and was directly used in the next reaction step. MS: 531 $(M+H)^+$, 1061 $(2M+H)^+$ (Figure S-9).

Preparation of Bis (3H-[1,2,3] triazolo[4,5-b] pyridin-3-yl) 3,3'-((2-((3-((3H-[1,2,3] triazolo[4,5-b] pyridin-3-yl) oxy)-3-oxopropoxy) methyl)-2-(6-(2,5-dioxo-2,5-dihydro-1H-pyrrol-1-yl) hexanamido) Propane-1,3-diyl) Bis(oxy)) Dipropionate (compound 13). Compound 12 was dissolved in 1 mL of dimethylformamide and 0.2 mL of N-methylmorpholine, then added to 114 mg (0.3 mmol) of HATU and stirred at RT for 2 h. The reaction solution was checked by mass spectrometer (Figure S-9) and was determined to be complete. This reaction solution was directly used in next reaction step. MS: 889 $(M+H)^+$, 985 $(M+H+NMM)^+$ (Figure S-10).

Preparation of Compound 14. A solution of compound 13 (0.2 mL, 0.017 mmol) was added to a solution of compound 7 (0.1 mmol) and stirred overnight at RT. It was checked by HPLC, then added dropwise to 0.5 mL (0.0425 mmol) of

compound 13 reaction solution. The reaction was monitored by HPLC and mass spectrometer until the reaction was over. The reaction solution was purified by HPLC to give 5 mg of pure product compound 14 (yield 6.0%). MS: (MALDI-TOF), 2482.6 $(M+H)^+$, 2504.6 $(M+Na)^+$ (Figure S-11) and (ESI), 1241.5 $(M+2H)^{2+}$ (Figure S-12).

Coupling Reaction. (a) Preparation of TCEP solution (10 mM): 2.9 mg (0.01 mmol) of TCEP was dissolved in 1 mL of water. The pH was adjusted to 7.0 by adding 1N sodium hydroxide solution. (b) Deprotection of the aptamer: 50 µL (200 µg) of 5-protected-thiol-PDGC-21 aptamer aqueous solution was added to 350 µL of TCEP solution (10 mM, pH=7) and shocked for 1 h at RT. The above reaction solution was purified by HPLC. The product was collected and dried by SpeedVac at 60°C. (c) Coupling aptamer with TNM-peptide-gemcitabine: The deprotected aptamer was dissolved in 300 µL of water. To this reaction solution was added TNM-peptide-gemcitabine (compound 14). Approximately 1.0 mg in 300 µL of DMF reacted under nitrogen protection at 4°C for 4 days. Then the reaction solution was monitored and purified by HPLC (Figure 2A) to give approximately 78 µg of Apt-TNM-peptide-gemcitabine based on UV absorbance at 260 nm.

Abbreviations

DCC: N,N'-Dicyclohexylcarbodiimide

DCM: Dichloromethane

DIPEA: N,N-Diisopropylethylamine

DMF: Dimethylformamide

EEDQ: 2-Ethoxy-1-ethoxycarbonyl-1,2-dihydroquinoline

HATU: (1-[Bis(dimethylamino)methylene]-1H-1,2,3-triazolo[4,5-b]pyridinium 3-oxide hexafluorophosphate

HPLC: High-performance liquid chromatography

MeOH: Methanol

NMM: N-Methylmorpholine

NHS: N-Hydroxysuccinimide

RT: Room temperature

TCEP: Tris(2-carboxyethyl)phosphine

TFA: Trifluoroacetic acid

TLC: Thin-layer chromatography

References

Yanming Wang, S. F. (2017). Development and Properties of Valine-Alanine based Antibody-Drug Conjugates with Monomethyl Auristatin E as the Potent Payload. Int J Mol Sci., 18(9), 1860.

Zihua Zeng, J. Q. (2021). Aptamers with Self-Loading Drug Payload and pH-Controlled Drug Release for Targeted Chemotherapy. Pharmaceutics, 13(8), 1221.

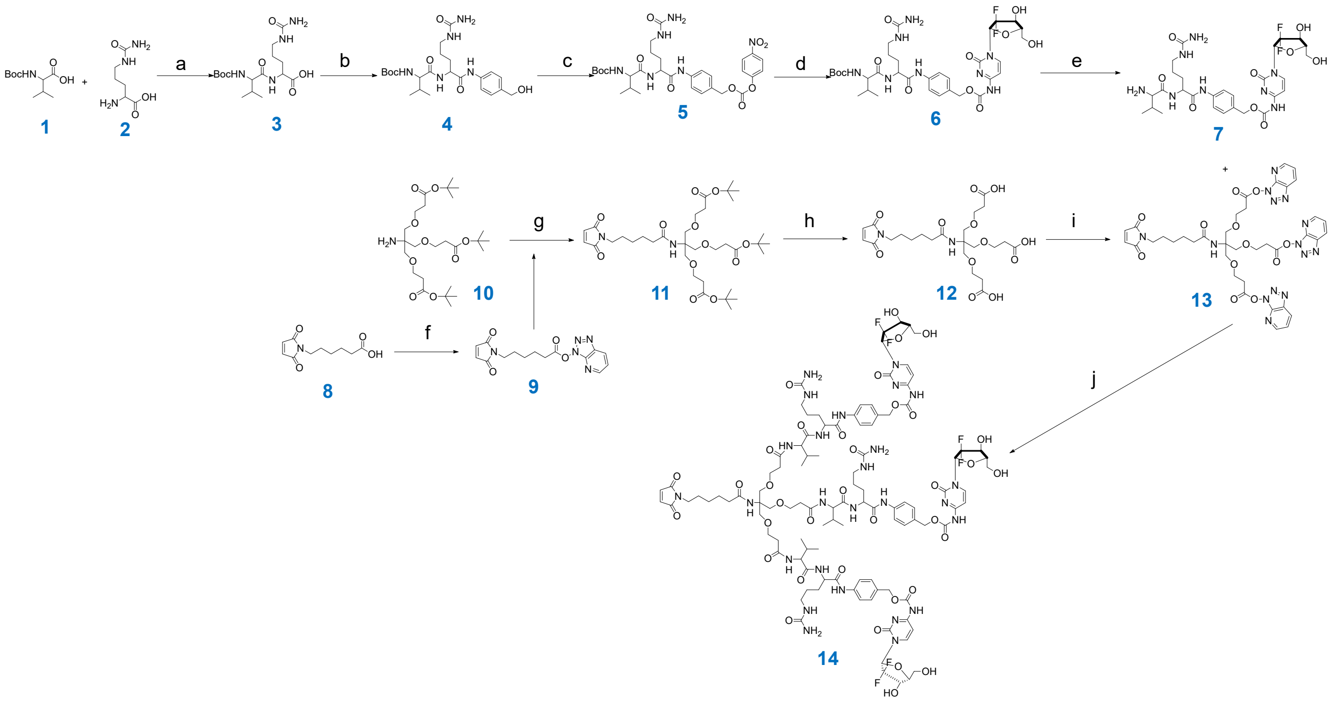

Scheme S1. Synthesis of TNM-linker-Gemcitabine structure. Reagents and condition: (a) DCM, NHS, DCC, RT 2 h; (b) DCM, MeOH, EEDQ, 4-Aminobenzyl alcohol, RT overnight; (c) DMF, DIEPA, 4-Nitrophenyl chloroformate, RT overnight; (d) DMF, DIEPA, Gemcitabine, RT 48 h; (e) TFA/DCM, RT 30 min; (f) DMF, NMM, HATU, RT 2 h; (g) RT 4 days; (h) MeOH, TFA, RT 1h; (i) DMF, NMM, HATU, RT 2 h; and (j) DMF, NMM, RT overnight.

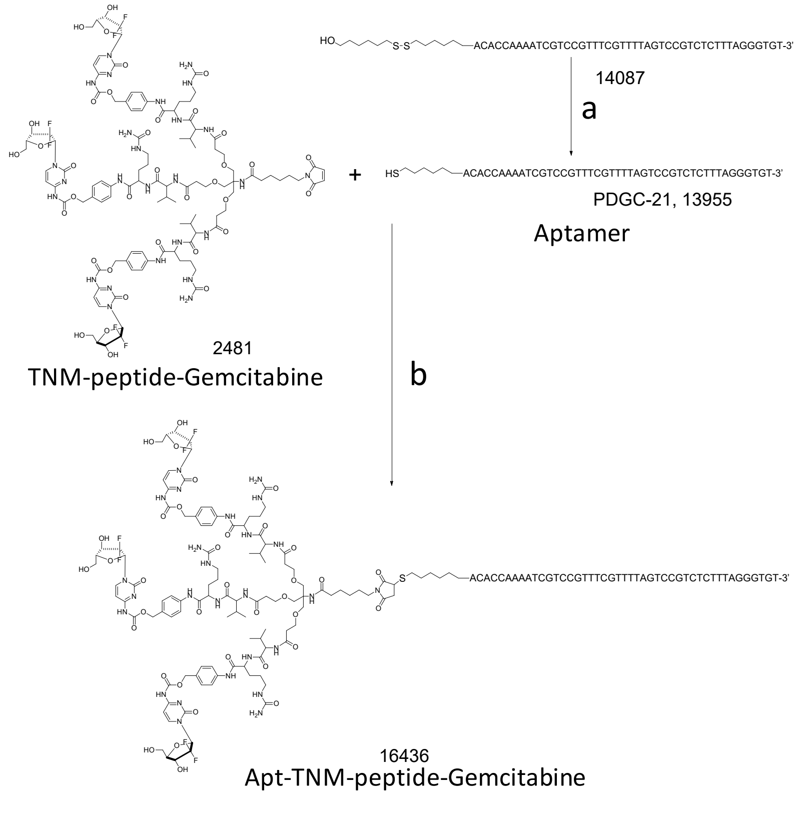

Scheme S2. Coupling reaction of aptamer with TNM-linker-Gemcitabine structure. Reagents and condition: (a)10 mM TCEP, RT 1 h; (b) water, 4°C, 4 days.

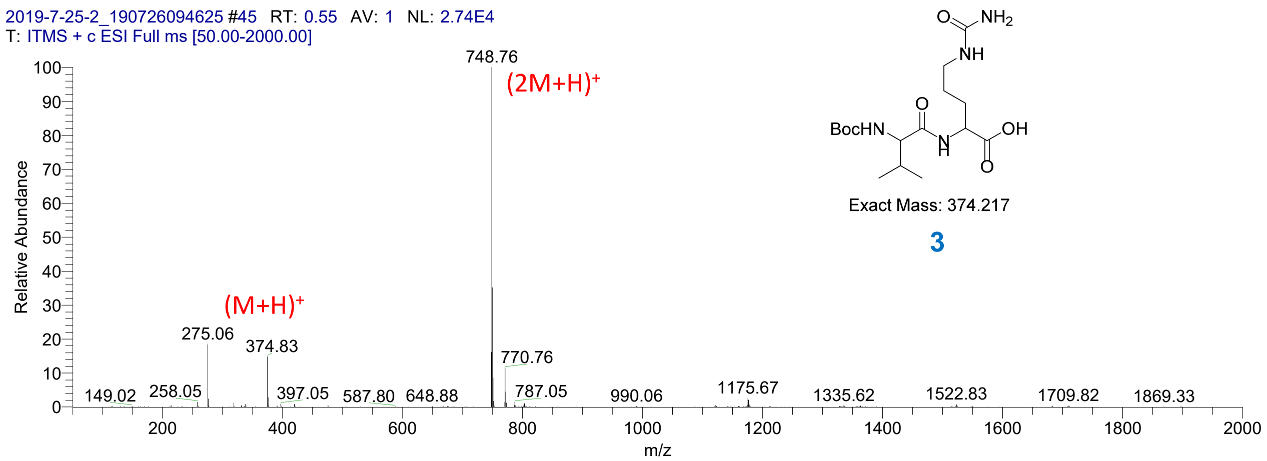

Figure S1. ESI mass spectrum of compound 3 in Scheme S-1. Mass spectrometry was performed on a Thermo LCQ FLEET Ion Trap Mass Spectrometer that was operated with electrospray ionization in the positive mode.

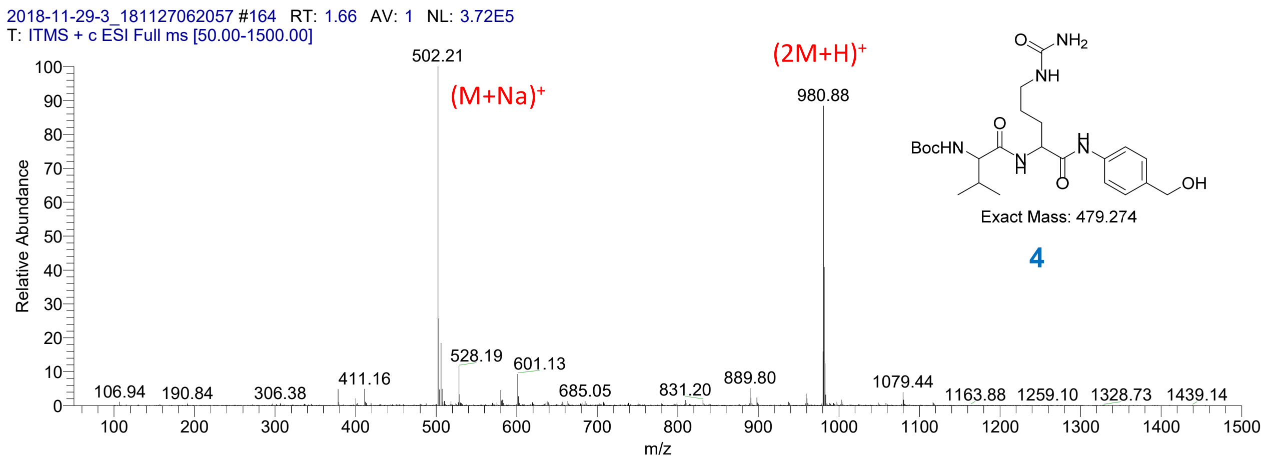

Figure S2. ESI mass spectrum of compound 4 Scheme S-1. Mass spectrometry was performed on a Thermo LCQ FLEET Ion Trap Mass Spectrometer that was operated with electrospray ionization in the positive mode.

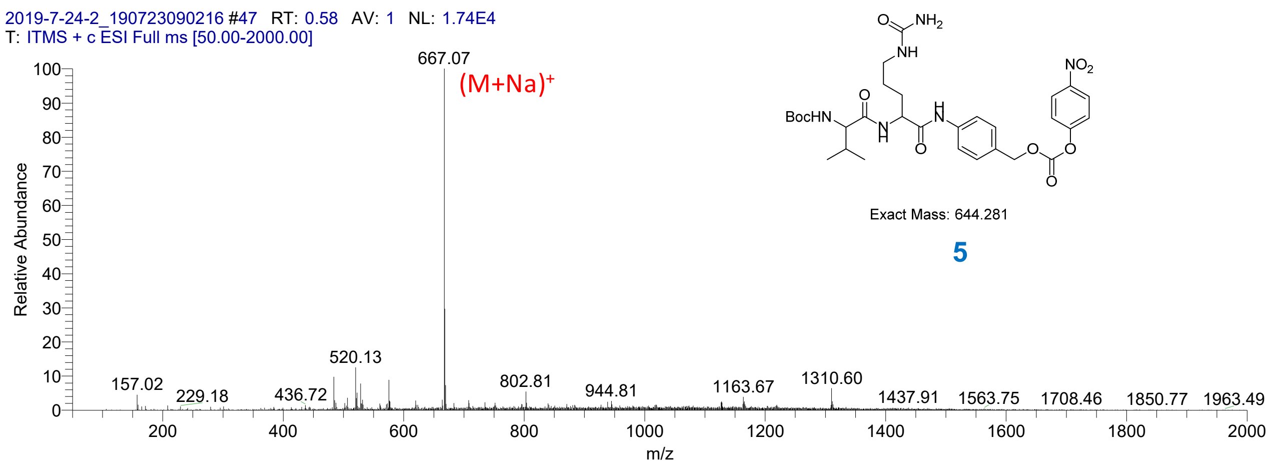

Figure S3. ESI mass spectrum of compound 5 Scheme S-1. Mass spectrometry was performed on a Thermo LCQ FLEET Ion Trap Mass Spectrometer that was operated with electrospray ionization in the positive mode.

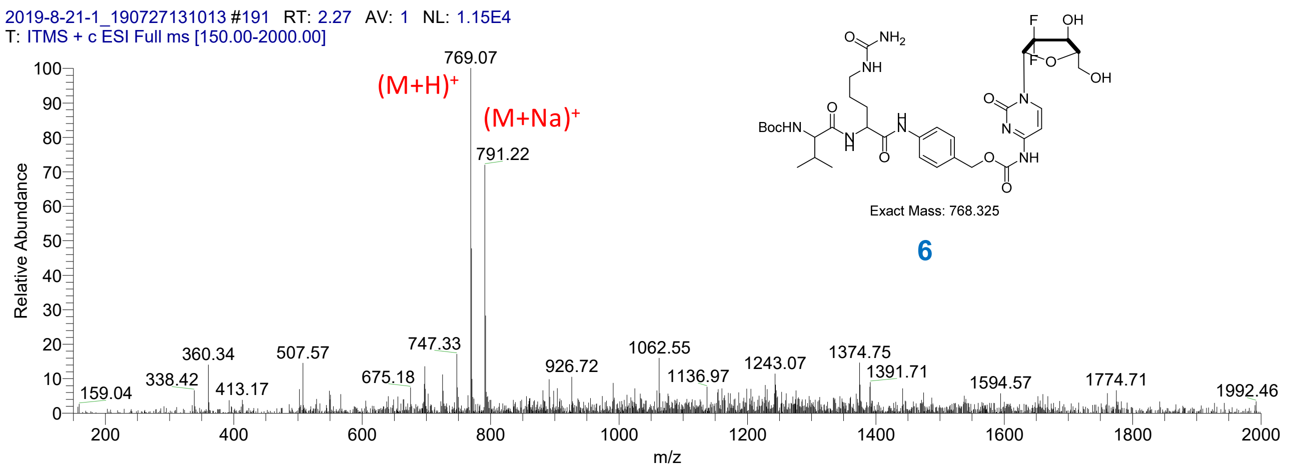

Figure S4. ESI mass spectrum of compound 6 Scheme S-1. Mass spectrometry was performed on a Thermo LCQ FLEET Ion Trap Mass Spectrometer that was operated with electrospray ionization in the positive mode.

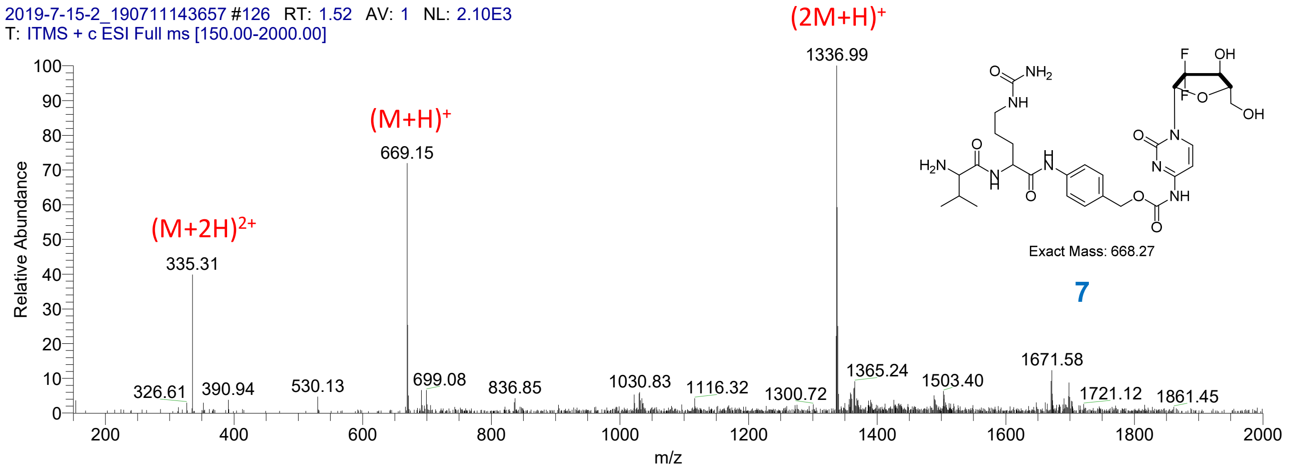

Figure S5. ESI mass spectrum of compound 7 Scheme S-1. Mass spectrometry was performed on a Thermo LCQ FLEET Ion Trap Mass Spectrometer that was operated with electrospray ionization in the positive mode.

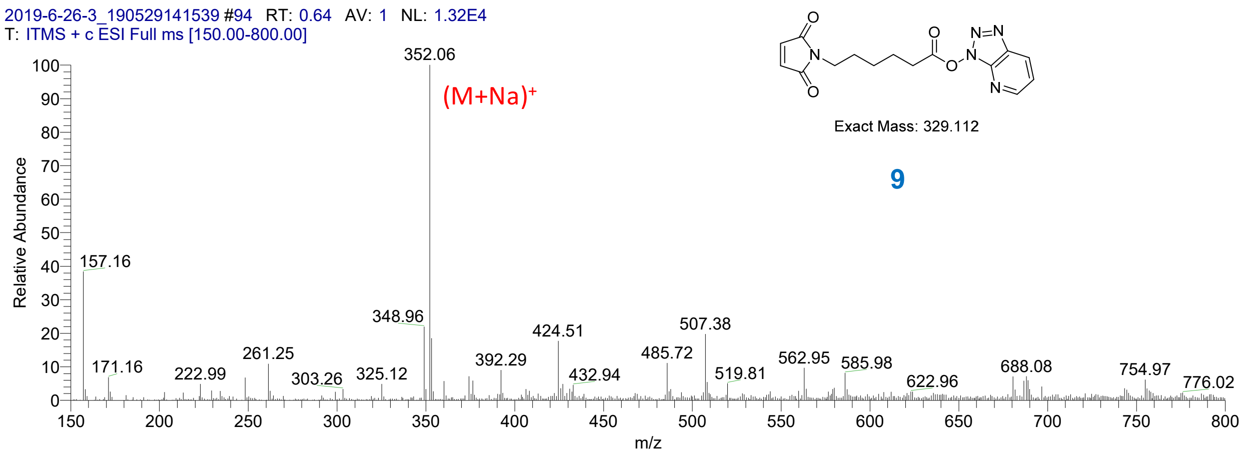

Figure S6. ESI mass spectrum of Compound 9 Scheme S-1. Mass spectrometry was performed on a Thermo LCQ FLEET Ion Trap Mass Spectrometer that was operated with electrospray ionization in the positive mode.

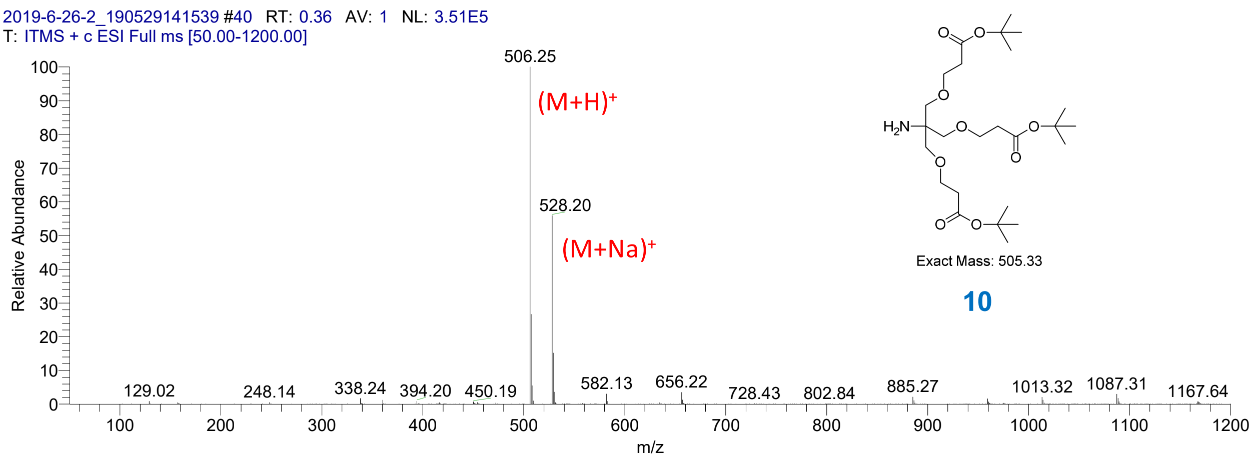

Figure S7. ESI mass spectrum of Compound 10 Scheme S-1. Mass spectrometry was performed on a Thermo LCQ FLEET Ion Trap Mass Spectrometer that was operated with electrospray ionization in the positive mode.

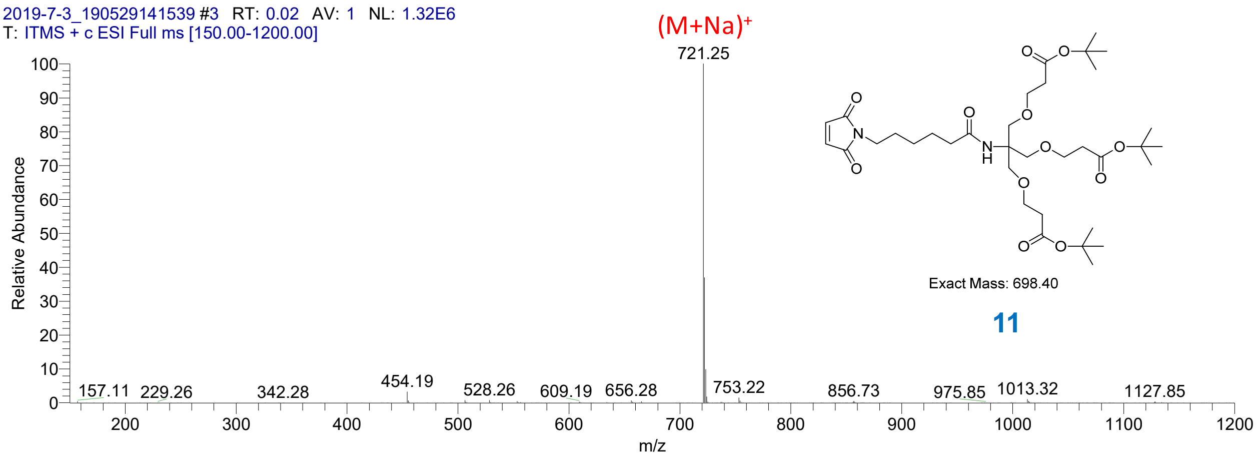

Figure S8. ESI mass spectrum of Compound 11 Scheme S-1. Mass spectrometry was performed on a Thermo LCQ FLEET Ion Trap Mass Spectrometer that was operated with electrospray ionization in the positive mode.

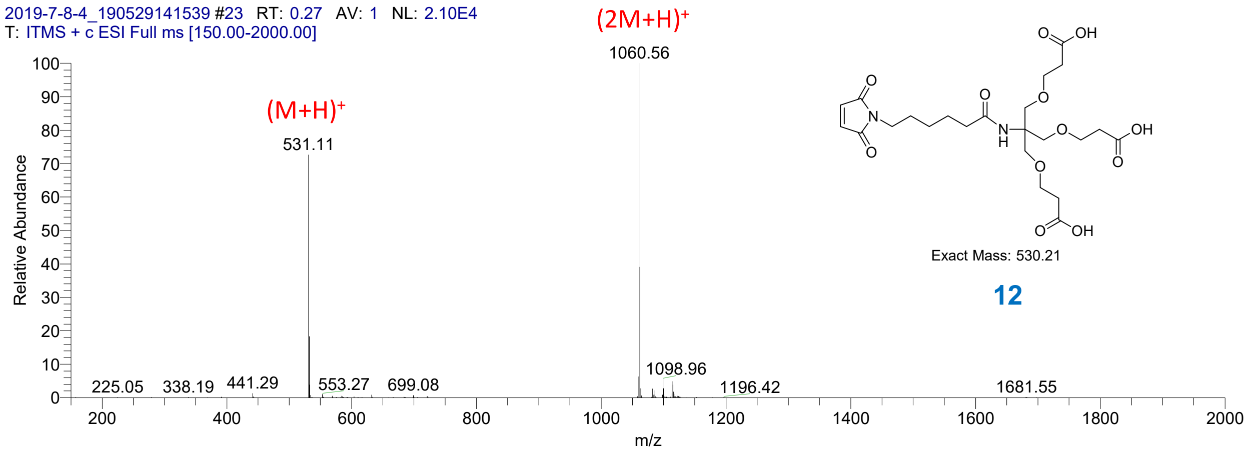

Figure S9. ESI mass spectrum of Compound 12 Scheme S-1. Mass spectrometry was performed on a Thermo LCQ FLEET Ion Trap Mass Spectrometer that was operated with electrospray ionization in the positive mode.

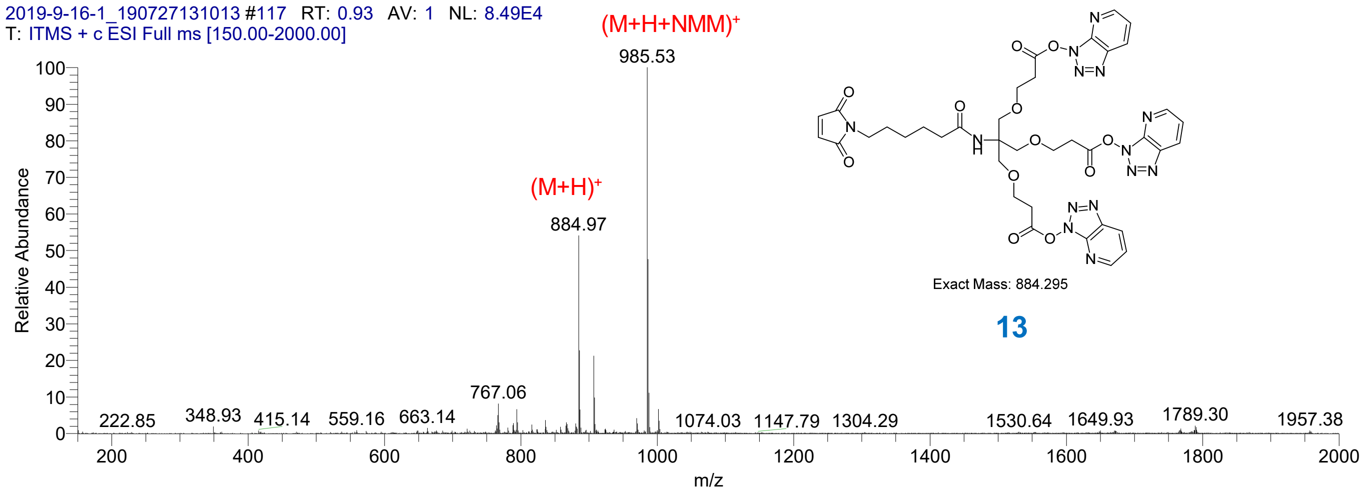

Figure S10. ESI mass spectrum of compound 13 Scheme S-1. Mass spectrometry was performed on a Thermo LCQ FLEET Ion Trap Mass Spectrometer that was operated with electrospray ionization in the positive mode.

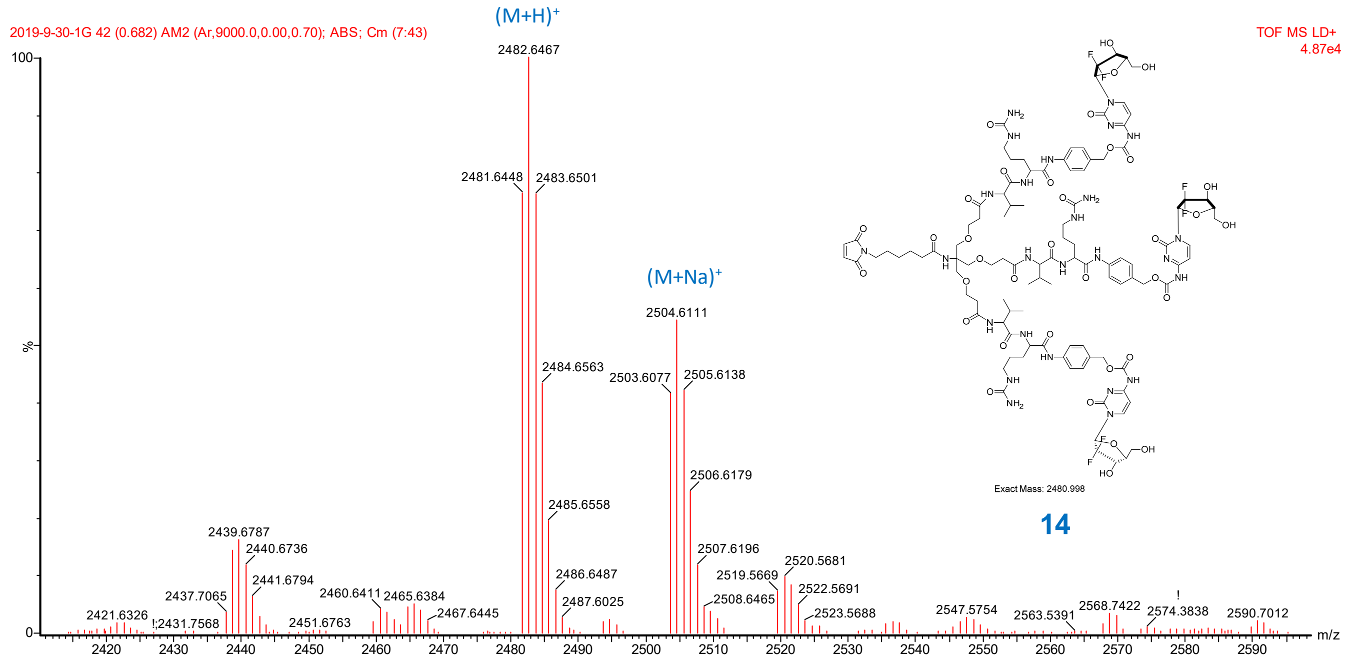

Figure S11. Maldi mass spectrum of compound 14 Scheme S-1. Mass spectrometry was performed on a Waters Maldi SYNAPT HDMS Q-TOF Mass Spectrometer that was operated with Maldi ionization in the positive mode.

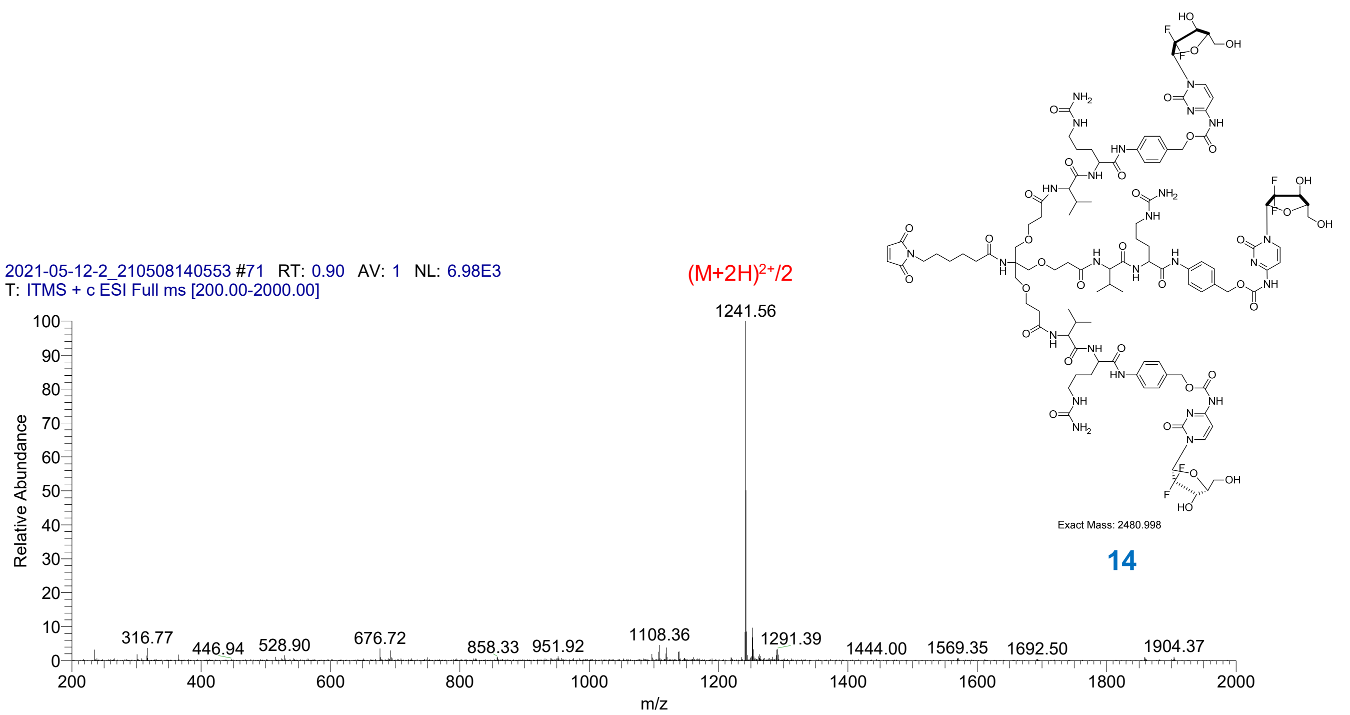

Figure S12. ESI mass spectrum of compound 14 Scheme S-1. Mass spectrometry was performed on a Thermo LCQ

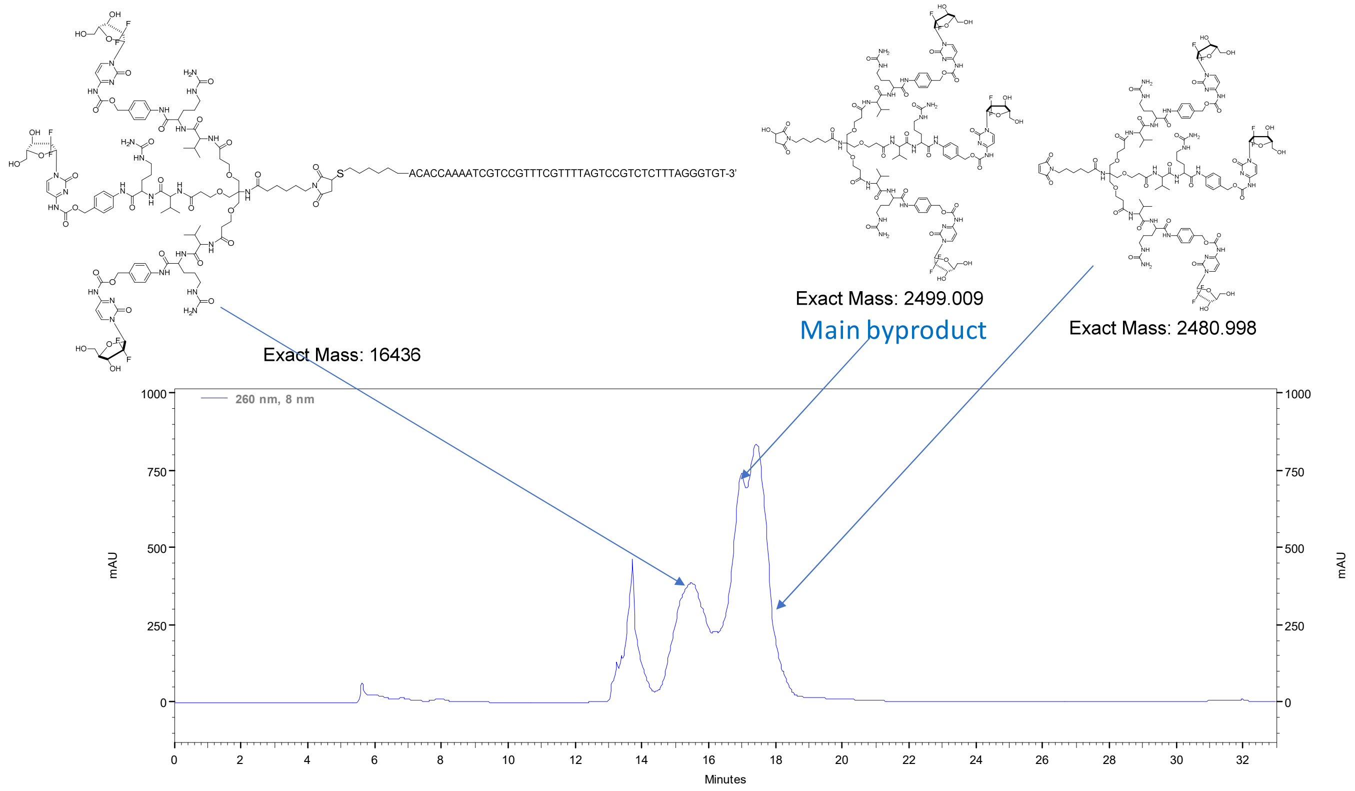

Figure S13. HPLC chromatogram. Purification of Apt-cL-triGemcitabine conjugate products by semi-preparative reversed-phase HPLC.

FLEET Ion Trap Mass Spectrometer that was operated with electrospray ionization in the positive mode.

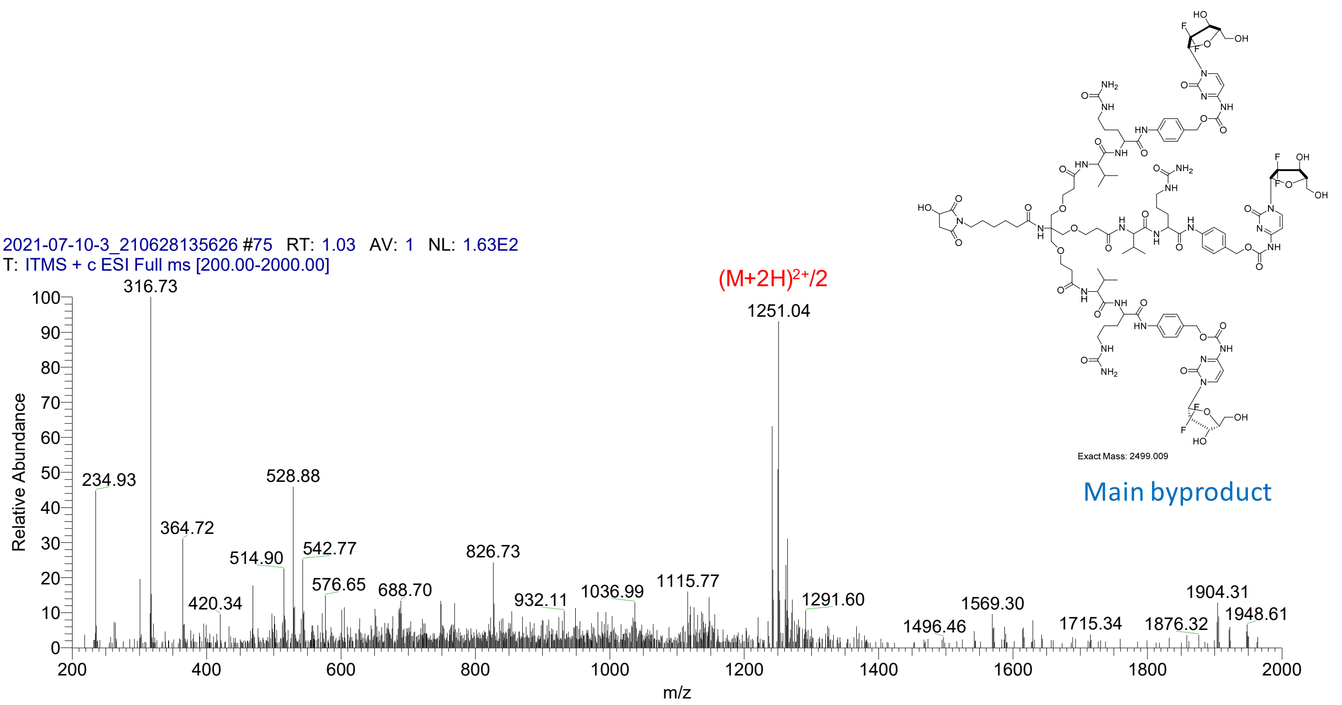

Figure S14. ESI mass spectrum of byproduct. Mass spectrometry was performed on a Thermo LCQ FLEET Ion Trap Mass Spectrometer that was operated with electrospray ionization in the positive mode.

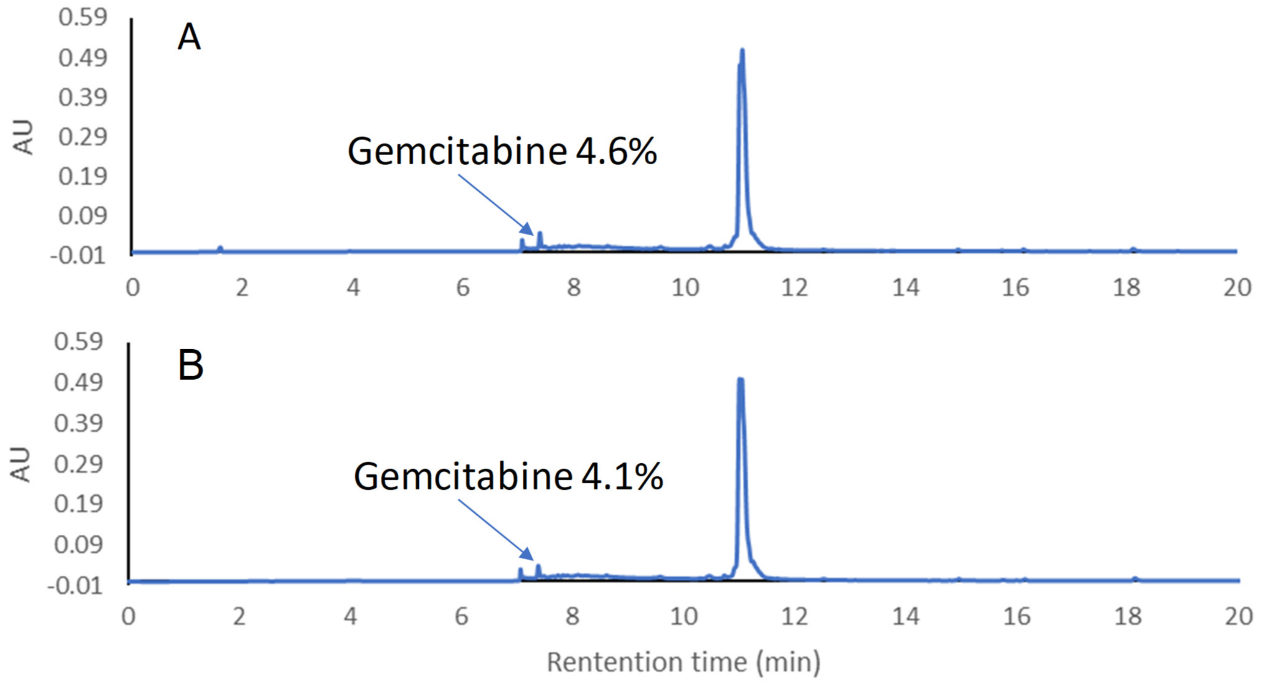

Figure S15. HPLC chromatogram of TNM-linker-Gemcitabine in different pH buffer. (A) TNM-peptide-Gemcitabine was incubated in pH=7.4 buffer at 37°C for 1 h; (B) TNM-peptide-Gemcitabine was incubated in pH=5.0 buffer 37°C for 1 h.

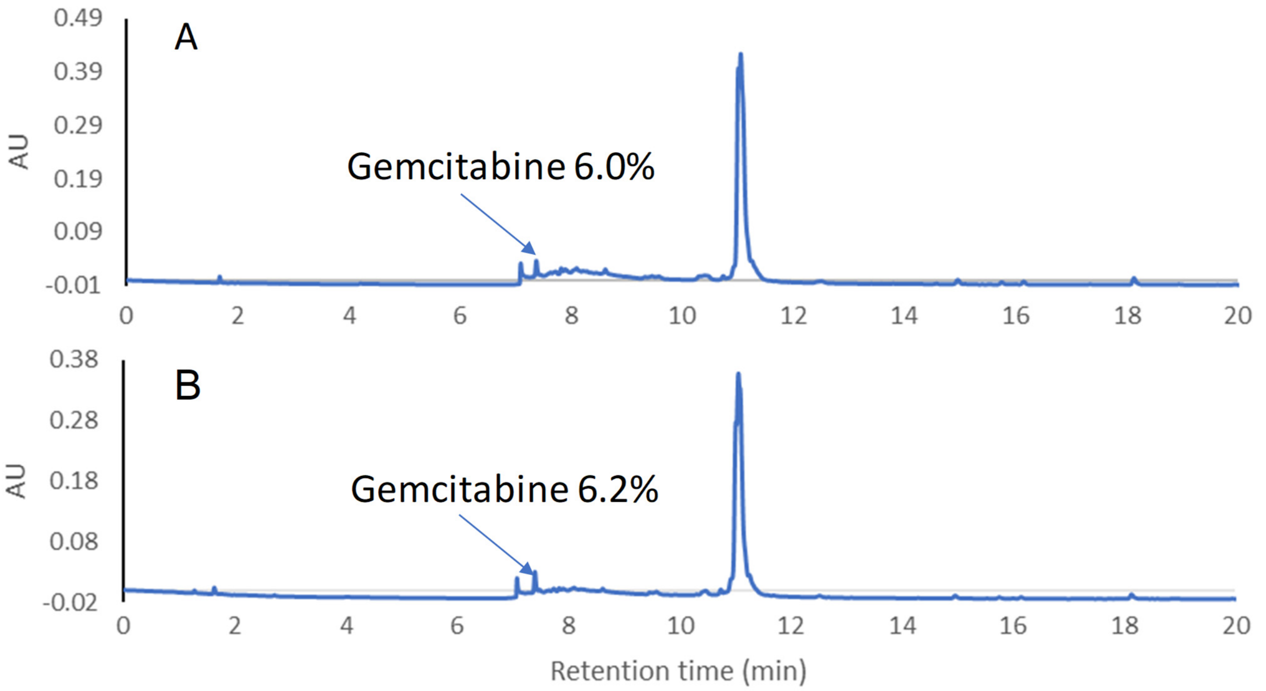

Figure S16. The chromatogram of TNM-linker-Gemcitabine structure post-treatment with inactive cathepsin B enzyme in pH=5 buffer at 37°C for 1 h. (A) TNM-peptide-Gemcitabine with inactivated cathepsin B (600: 1 mole ratio). (B) TNM-peptide-Gemcitabine with inactivated cathepsin B (800:1 mole ratio).

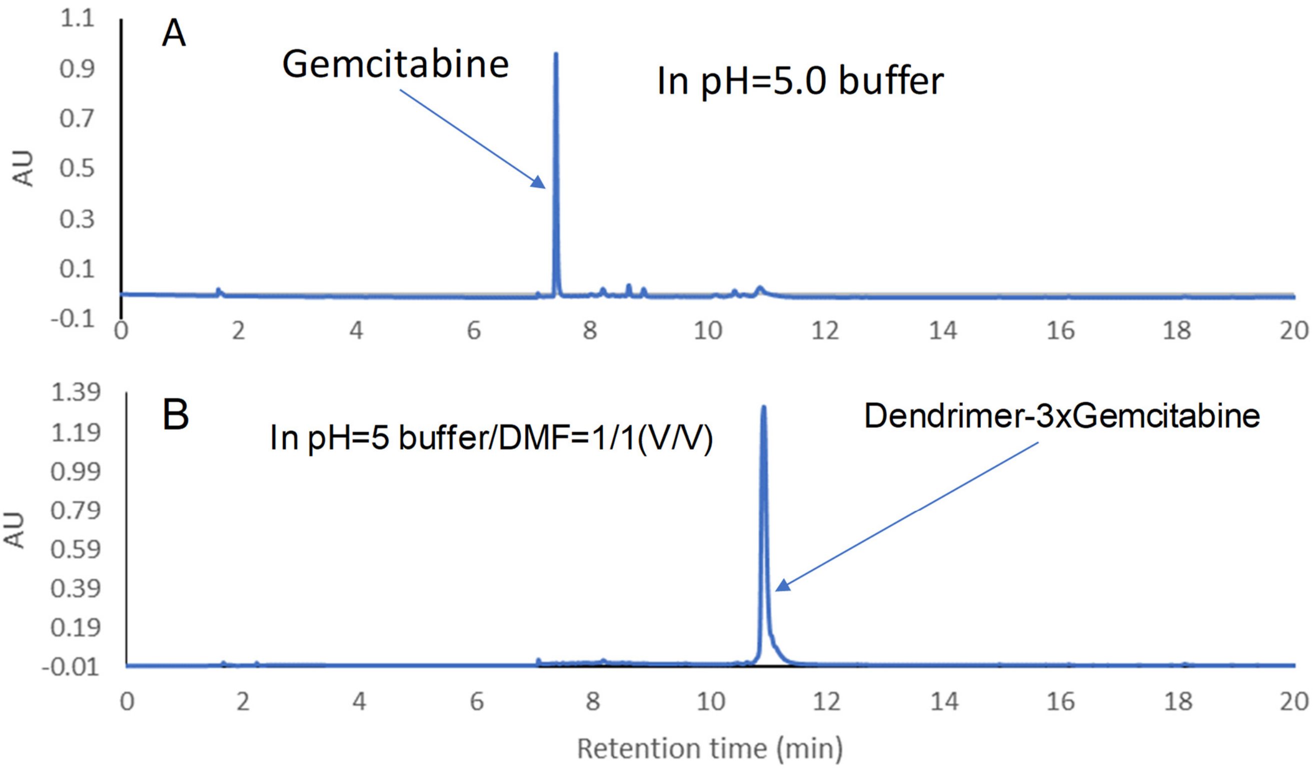

Figure S17. The chromatogram of TNM-liker-Gemcitabine structure treated with activated cathepsin B enzyme in pH=5 buffer for 1 h at 37°C. (A) In pH=5 buffer. (B) In pH=5 buffer/DMF=1/1(V/V).

Table S1. Apoptosis rates and dead cell rates two days after treatment

MDA-MB-231 cell		Annexin V positive (%) (SD)	PI positive (%) (SD)
Apt-cL-TriGemcitabine	0 nM	1.03 (0.58)	0.83 (0.44)
	100 nM	5.38 (2.54)	2.30 (0.78)
	250 nM	17.02 (5.90)	5.70 (2.07)
Free Gemcitabine	0 nM	1.03 (0.58)	0.83 (0.44)
	100 nM	3.10 (0.63)	1.48 (0.31)
	250 nM	3.95 (1.82)	1.55 (0.57)

T47D cell		Annexin V positive (%) (SD)	PI positive (%) (SD)
Apt-cL-TriGemcitabine	0 nM	1.53 (0.31)	2.24 (0.62)
	100 nM	2.82 (1.85)	2.44 (1.07)
	250 nM	3.03 (0.41)	2.47 (0.88)
Free Gemcitabine	0 nM	1.53 (0.31)	2.24 (0.62)
	100 nM	5.94 (2.88)	5.51 (2.65)
	250 nM	33.39 (6.17)	25.41 (3.73)

NOTE: Apoptotic cells are stained by Annexin V and dead cells by PI. SD = Standard Deviation.
